# Splicing targeting drugs highlight intron retention as an actionable vulnerability in advanced prostate cancer

**DOI:** 10.1186/s13046-024-02986-0

**Published:** 2024-02-27

**Authors:** Chiara Naro, Ambra Antonioni, Vanessa Medici, Cinzia Caggiano, Ariane Jolly, Pierre de la Grange, Pamela Bielli, Maria Paola Paronetto, Claudio Sette

**Affiliations:** 1 Department of Neuroscience, Section of Human Anatomy, Catholic University of the Sacred Heart, Largo Francesco Vito 1, 00168 Rome, Italy; 2 GSTeP Organoids Research Core Facility, Fondazione Policlinico Universitario A. Gemelli, IRCCS, 00168 Rome, Italy; 3 GenoSplice, Paris, France; 4 Department of Biomedicine and Prevention, University of Rome Tor Vergata, 00133 Rome, Italy; 5 Laboratory of Molecular and Cellular Neurobiology, IRCCS Fondazione Santa Lucia, 00143 Rome, Italy; 6 University of Rome Foro Italico, 00135 Rome, Italy

**Keywords:** Advanced prostate cancer, Splicing inhibitors, Alternative splicing, Intron-retention, 3’-end mRNA processing, Transcriptomics

## Abstract

**Background:**

Advanced prostate cancer (PC) is characterized by insensitivity to androgen deprivation therapy and chemotherapy, resulting in poor outcome for most patients. Thus, advanced PC urgently needs novel therapeutic strategies. Mounting evidence points to splicing dysregulation as a hallmark of advanced PC. Moreover, pharmacologic inhibition of the splicing process is emerging as a promising option for this disease.

**Method:**

By using a representative androgen-insensitive PC cell line (22Rv1), we have investigated the genome-wide transcriptomic effects underlying the cytotoxic effects exerted by three splicing-targeting drugs: Pladienolide B, indisulam and THZ531. Bioinformatic analyses were performed to uncover the gene structural features underlying sensitivity to transcriptional and splicing regulation by these treatments. Biological pathways altered by these treatments were annotated by gene ontology analyses and validated by functional experiments in cell models.

**Results:**

Although eliciting similar cytotoxic effects on advanced PC cells, Pladienolide B, indisulam and THZ531 modulate specific transcriptional and splicing signatures. Drug sensitivity is associated with distinct gene structural features, expression levels and cis-acting sequence elements in the regulated exons and introns. Importantly, we identified PC-relevant genes (i.e. *EZH2*, *MDM4*) whose drug-induced splicing alteration exerts an impact on cell survival. Moreover, computational analyses uncovered a widespread impact of splicing-targeting drugs on intron retention, with enrichment in genes implicated in pre-mRNA 3’-end processing (i.e. *CSTF3*, *PCF11*). Coherently, advanced PC cells displayed high sensitivity to a specific inhibitor of the cleavage and polyadenylation complex, which enhances the effects of chemotherapeutic drugs that are already in use for this cancer.

**Conclusions:**

Our study uncovers intron retention as an actionable vulnerability for advanced PC, which may be exploited to improve therapeutic management of this currently incurable disease.

**Supplementary Information:**

The online version contains supplementary material available at 10.1186/s13046-024-02986-0.

## Background

Alternative splicing (AS) allows production of multiple transcript and protein variants from most human genes [1, 2]. Changes in epigenetic chromatin marks, transcription elongation rate and signal transduction pathways were all shown to affect the recruitment of splicing factors and/or the activity of the core splicing machinery—the spliceosome—thus allowing cells to adapt to environmental changes through AS regulation [3–5]. Nevertheless, the extreme flexibility of AS, which is based on the degenerate nature of the sequences defining the exon–intron junctions, is prone to errors and splicing defects have been associated with many human diseases, including cancer [6–9]. Indeed, most human cancers display highly dysregulated splicing patterns, which result in the production of oncogenic protein isoforms or in the functional disruption of tumour suppressor proteins [6, 7, 9].

Aberrant splicing regulation can result from mutations in core spliceosome genes, as in the case of the gene encoding for the splicing factor 3B1 (*SF3B1*) in hematological malignancies [6, 7, 9], or from up-regulation of splicing factors, which alter the physiological balance between positive and negative regulators of exon recognition by the spliceosome [10]. A crucial driver of splicing dysregulation in human cancer is the oncogenic transcription factor MYC [11]. Amplification or up-regulation of MYC leads to a global increase in the transcriptional output of cells, thus imposing a burden on the spliceosome and causing pervasive intron retention (IR) and aberrant selection of splice sites (ss) [12]. Furthermore, MYC drives transcription of oncogenic splicing factors that alter splicing decisions in cancer cells and promote oncogenic pathways [13–16]. However, while splicing dysregulation generally confers an advantage to cancer cells, it also represents a therapeutically exploitable vulnerability [6, 9, 17]. For instance, splicing defects generate neoepitopes that can be targeted by immunotherapeutic approaches [18]. Moreover, *SF3B1* mutations sensitize cancer cells to splicing-targeting drugs, such as inhibitors of the spliceosome or of kinases that affect RNA processing [7, 9, 19]. Thus, understanding the mechanisms of splicing dysregulation in cancer cells and evaluating the efficacy of splicing targeting drugs can pave the ground for novel therapeutic approaches to cancer [6, 7].

One cancer type that is particularly dependent on splicing regulation is advanced prostate cancer (PC). PC cells require signalling from the androgen receptor (AR) to proliferate and deprivation of androgens represents the first line treatment for patients with advanced disease [20]. However, after initial remission, PC generally evolves to a castration-resistant (CR) stage for which current therapies are poorly effective [21]. Defective splicing of the AR pre-mRNA yields truncated protein variants (AR-Vs) that are constitutively active and insensitive to anti-hormonal therapies [22], thus contributing to disease progression. Expression of AR-Vs in castration-resistant prostate cancer (CRPC) samples was correlated with that of MYC [23], which is also frequently amplified in PC [24]. MYC amplification affects expression of multiple splicing factors and causes widespread AS changes in primary PC tissues and cell lines [24]. Notably, the severity of splicing dysregulation correlates with PC progression and pervasive IR was suggested as a hallmark of disease that associates with stem-like properties and malignancy [25]. At the same time, treatment with a spliceosome inhibitor was effective in reversing CRPC malignancy [25], suggesting that splicing regulation represents a therapeutically actionable vulnerability for advanced PC.

In this study, we have explored the genome-wide impact exerted on CRPC cells by three drugs that inhibit splicing at different steps. Pladienolide B (PladB) directly binds to and impairs the activity of SF3B1, a core protein of the U2 small nuclear ribonucleoprotein (snRNP) that recognize the 3' ss at early stages of the splicing reaction [26]. Indisulam acts as molecular glue to promote the interaction of the ubiquitin ligase DCAF15 with the U2 auxiliary factor of 65 kDa (U2AF65)-like protein RBM39, leading to its proteasome-mediated degradation [27]. THZ531 inhibits CDK12 and CDK13, which phosphorylate the RNA polymerase II (RNAPII) and couple transcriptional elongation with pre-mRNA processing regulation [19]. All three inhibitors strongly impaired proliferation and survival in 22Rv1 cells, a well-established CRPC cell line. However, while the cytotoxic effects of these drugs are similar, the signature of genes affected at splicing and expression level is largely distinct. Bioinformatics analyses highlighted common and unique features of exons and introns modulated by the three drugs. Moreover, we identified several PC-relevant genes (i.e. *EZH2*, *MDM4*, *AR*, *ERBB3*, *CSTF3*, *PCF11*) whose splicing alteration exerts an impact on CRPC cell survival. Thus, our findings uncover actionable vulnerabilities that are induced by pharmacologic dysregulation of splicing in CRPC cells and highlight new therapeutic targets that can be exploited to treat this currently incurable disease.

## Methods

### Cell culture, treatments, proliferation and cytotoxic assay

LNCaP and 22Rv1 cells were grown in RPMI 1640 (Euroclone), supplemented with 4500 mg/l glucose (Sigma-Aldrich), 10 mM HEPES, 1 mM sodium pyruvate (Euroclone) and 10% fetal bovine serum (Gibco, Life Technologies). For androgen deprivation conditions, cells were grown in medium containing 10% charcoal-stripped serum (CSS) for 24 h before treatment and during the treatment. Cells were grown in a 37 °C humidified atmosphere of 5% CO_2_. 24 h post seeding cells were treated with the indicated drugs (Additional File 1: Supplemental Table 1) and harvested at indicated time points for protein and/or RNA analysis. Proliferation assays were performed with the IncuCyte SX5 Live-content imaging system (Essen Bioscience) by measuring either cell confluence or number of live cells. For cytotoxicity assays, dying cells were labelled with the Cytotox Green Dye (Sartorius), and Nuclight Rapid NIR (Sartorius) was used to label nuclei of all cells. At least four images/well were acquired and analysed using the IncuCyte Cell-by-Cell software.

### Protein extracts and western blot analysis

Protein extracts were prepared as previously described [28]. Briefly, cells were lysed in [30 mM Tris pH 7.5; 100 mM NaCl; 10 mM MgCl2; 1% Triton X-100; 1 mM DTT; 0,5 mM Na3VO4; protease inhibitor cocktail (Sigma Aldrich)], briefly sonicated, incubated on ice for 20 min and centrifuged for 20 min at 12,000 g at 4 °C. Protein extracts were then analysed by Western Blot using the following primary antibodies: rabbit anti-HER3/ErbB3 (D22C5) (mAb#12,708, Cell Signaling), anti- AR (06–680, Millipore), anti-PCF11 (A303-706A, Bethyl Laboratories), anti-CSTF3 (A301-094, Bethyl Laboratories), anti-RBM39 (HPA001591, Atlas Antibodies), anti-H3 (Ab1791, Abcam), anti-H3K27me3 (mAb#9733, Cell Signaling); mouse anti-HSP90 (sc-13119, Santa Cruz), anti-ACTIN (sc-47778 Santa Cruz), anti-EZH2 (AC22) (mAb#3147, Cell Signaling), anti-MDM4 (clone 7A8, 04–1556, Sigma Aldrich). Anti-rabbit, anti-mouse (GE Healthcare) HRP-linked secondary antibodies were all used at 1:5000 dilution and ECL signal developed using Clarity Western ECL Substrates (Biorad) or Amersham ECL Select™. Densitometric analyses were performed using Alliance system software (UVITEC, Cambridge).

### Extraction of RNA, RT-PCR and real-time PCR analysis

Total RNA was extracted and DNAse-treated using the RNA extraction GeneAid (GeneAid Biotech) kit. 250 ng – 1 mg of total RNA was retro-transcribed with oligo-dT primers, using M-MLV reverse transcriptase (Promega). cDNA was used as template for PCR (GoTaq, Promega) and reactions were analysed on agarose or acrylamide gels. Densitometric analyses were performed using the Image Lab software (Biorad). Quantitative real-time PCRs (RT-qPCR) were performed using LightCycler 480 SYBR Green I Master and the LightCycler 96 System (Roche), according to the manufacturer’s instructions. Control reactions omitting M-MLV reverse transcriptase were also carried out. All primers used are listed in Additional File 1: Supplementary Table S2.

### RNA SEQ and analysis

For RNA-seq analysis, 22Rv1 cells were treated with indisulam (3.3 µM, 12 h), or Pladienolide B (10 nM, 6 h) or THZ531 (200Nm, 6 h) and total RNA extracted and DNAse treated using the RNEasy mini kit (Qiagen), according to manufacturer’s instruction. PolyA-plus RNA-seq libraries were constructed according to Illumina’s protocol and sequenced using a 100 bp paired-end format. RNA-Seq data analysis was performed as previously described [29, 30]. Results were considered statistically significant for *p*-values ≤ 0.05 and fold-changes ≥ 2. RNA-Seq data are available on GEO (NCBI) under accession GSE234734.

### Bioinformatic analysis

Clinical and transcriptomic data from the TGCA project [31] were downloaded and analysed using the UCSC Xena platform [32]. Kaplan Meier analyses were performed using the online KM plotter tool [33]. Analysis of 3’ and 5′ splice-sites strength, GC context, polypyrimidine tract length/score, distance of branchpoint from acceptor splice-site, median expression levels of regulated genes were evaluated as previously described [29, 30, 34]. Functional annotation analyses were performed using the online Enrichr gene set search tool [35].

### siRNA and antisense oligonucleotides transfection

For RNA interference, cells were transfected with control (5′-AGACGAACAAGUCACCGAC-3′) or PCF11 (5′-CGACAGCUAUUUCAGUAUCAATT-3′) targeting siRNAs (Sigma- Aldrich) using Lipofectamine RNAiMAX (Invitrogen) and harvested 48 h later for protein and RNA analyses. For antisense oligonucleotide (ASO) transfection cells were transduced by scraping delivery according to the manufacturer’s instructions (Gene Tools) with 10 µM of MDM4 exon 7 targeting ASO (5'- CGTGTGGTGATTTTACCTTCAGTTG -3') or control ASO (5'-TCATTTGCTTCATACACAGG-3').

### Statistical analysis

Statistical analyses for differential gene expression, splicing changes, analysis of structural features of regulated genes and exons were performed in R using the statistical tests described in the figure legends. Statistical analyses for cell proliferation and cytotoxicity assays, qRT-PCR, densitometric analysis of PCR and Western blot results were performed in GraphPad Prism using the statistical tests described in the figure legends. Number of replicates independently analysed is indicated in each figure legend. Results were considered statistically significant if *p*-value ≤ 0.05, *p ≤ 0.05, **p ≤ 0.01, ***p ≤ 0.001; ****p ≤ 0.0001; ns = not significant.

## Results

### Splicing targeting drugs extensively modulate the transcriptome of CRPC cells

CRPC is resistant to anti-androgen therapy. Although new generation AR inhibitors, like enzalutamide, exhibit enhanced efficacy, their clinical benefit remains limited [20, 21]. The 22Rv1 cell line is an established model of the CRPC stage, which maintains insensitivity to Enzalutamide with respect to the androgen dependent LNCaP cell line (Additional file 2: Fig. S1A). Thus, we employed 22Rv1 cells to evaluate the efficacy of indisulam, PladB and THZ531. All three splicing targeting drugs efficiently suppressed growth of 22Rv1 cells, with IC_50_ values in the nanomolar range for PladB (1.9 nM) and THZ531 (109.6 nM) and in the low micromolar range for indisulam (6.8 µM) (Additional File 2: Fig. S1B), with no significant changes when cells were maintained in androgen depleted (CSS) medium (Additional File 2: Fig. S1C). Growth inhibition was significantly detected after 48 h (hrs) of treatment, while it reached an almost complete block at 96 h of treatment with 3.3 µM indisulam, 10 nM PladB and 200 nM THZ531 (Additional File 2: Fig. S1). At these concentrations, all three drugs significantly induced cell death at 48 h of treatment (Additional File 2: Fig. S1E and F).

**Fig. 1:**
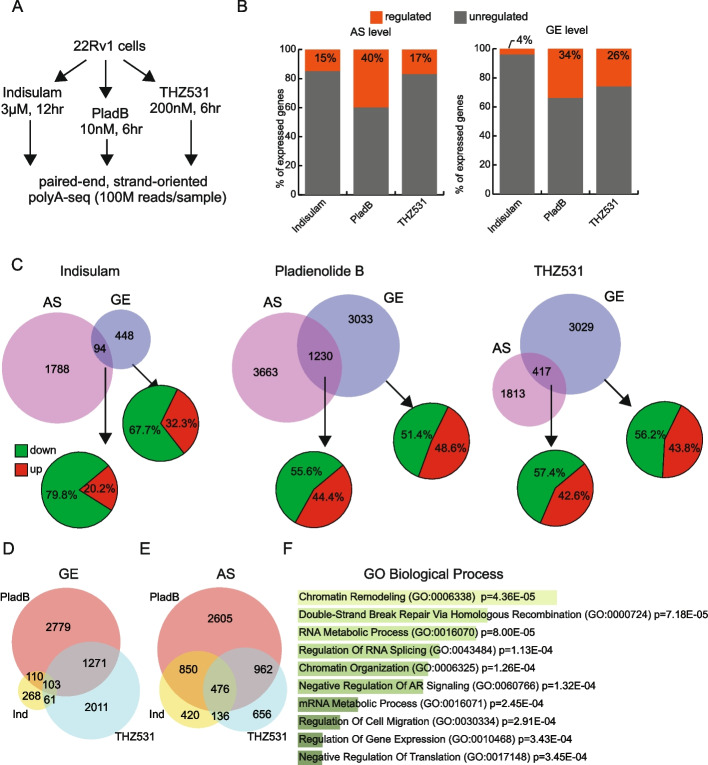
Alternative-splicing and expression changes elicited in 22Rv1 cells by treatment with Indisulam, Pladienolide B and THZ531. **A** Schematic representation of the experimental workflow and RNA-seq analysis performed in 22Rv1cells (PladB, pladienolide B). **B** Bar graphs representing the % of genes of 22Rv1 transcriptome regulated at either alternative splicing (AS, left panel) or gene-expression (GE, right panel) level. **C** Venn diagrams showing the overlap between genes regulated at AS and GE level by indicated treatment. For GE-regulated genes, either exclusively or in the overlap with AS, % of up- and down-regulated genes are indicated. **D**, **E** Venn diagrams showing the overlap for GE (**D**) and AS (**E**) regulated genes by the indicated drugs in 22Rv1 cells respect to control. **F** Bar graph illustrating the GO terms relative to biological processes significantly enriched within the AS regulated genes by all drugs in (**E**) (analysis performed with Enrichr tool, *p*-value < 0,05)

To evaluate the effects of these drugs on the transcriptome, 22Rv1 cells were treated with optimal doses of PladB (10 nM) and THZ531 (200 nM) for 6 h and of indisulam (3 µM) for 12 h (Fig. 1A). Indisulam was administered for extended time to allow complete degradation of RBM39 (Additional File 2: Fig. S1G,H). RNA sequencing analyses documented that all three drugs caused widespread changes in the transcriptome (fold difference > 2, *p* < 0.05; Additional File 3: Supplementary Tables S3-S8), with PladB affecting the largest percentage of genes both at splicing and expression level, followed by THZ531 and indisulam (Fig. 1B). In line with their direct inhibition of spliceosomal factors [26, 27], indisulam and PladB regulated a larger number of genes at AS level (Fig. 1B), whereas inhibition of the transcriptional kinases CDK12 and CDK13 by THZ531 was more effective at gene expression (GE) level (Fig. 1B). While several genes were affected by treatments at both GE and AS level (GE/AS), the majority displayed a specific regulation by the three drugs (Fig. 1C). At GE level, indisulam preferentially downregulated gene expression (67,7% for GE-only and 79,8% for GE/AS), whereas the genes affected by PladB and THZ531 were more equally distributed between up- and down-regulated (Fig. 1C). Our analysis also highlighted hundreds of genes regulated at the AS level by all three drugs, whereas fewer genes were co-regulated at GE level (Fig. 1D,E; Supplementary Tables S3-S8). Common splicing-regulated genes (*n* = 476) were enriched in biological processes with strong relevance for PC, such as chromatin remodelling, DNA repair, RNA metabolism and regulation of AR signalling (Fig. 1F, Additional File 4: Supplementary Table S9). These results indicate that short treatment with RNA processing inhibitors elicits widespread and multilayered effects on the transcriptome of CRPC cells.

### Structural properties of the genes regulated by splicing targeting drugs

Since indisulam, PladB and THZ531 regulated both common and specific gene subsets in CRPC cells (Fig. 1D and E), we searched for structural features underlying the sensitivity to each of these drugs. Among the GE-only genes, indisulam (*p* = 1,06^e−05^) and PladB (*p* = 2,35^e−72^) caused the up-regulation of particularly small genes (Additional File 2: Fig. S2A). Furthermore, genes up-regulated by these drugs were characterized by an extremely low mean intron length (Additional File 2: Fig. S3A) and median intron/exon ratio (Additional File 2: Fig. S3B), suggesting the lack of introns. Accordingly, manual inspection revealed that many of these genes are intronless, such as those encoding small nucleolar RNAs (SNORs) or other unannotated single exon genes (Additional File 2: Fig. S3C,D; Additional File 3: Table S4-S8). This result suggests that targeting the activity of the spliceosome in CRPC cells redirects the RNAPII complex to small genes, which may be transcribed more efficiently because are less dependent on intron removal. On the other hand, genes down-regulated in the presence of both spliceosome inhibitors were mainly characterized by a low expression level in 22Rv1 cells (Additional File 2: Fig. 2B).

**Fig. 2:**
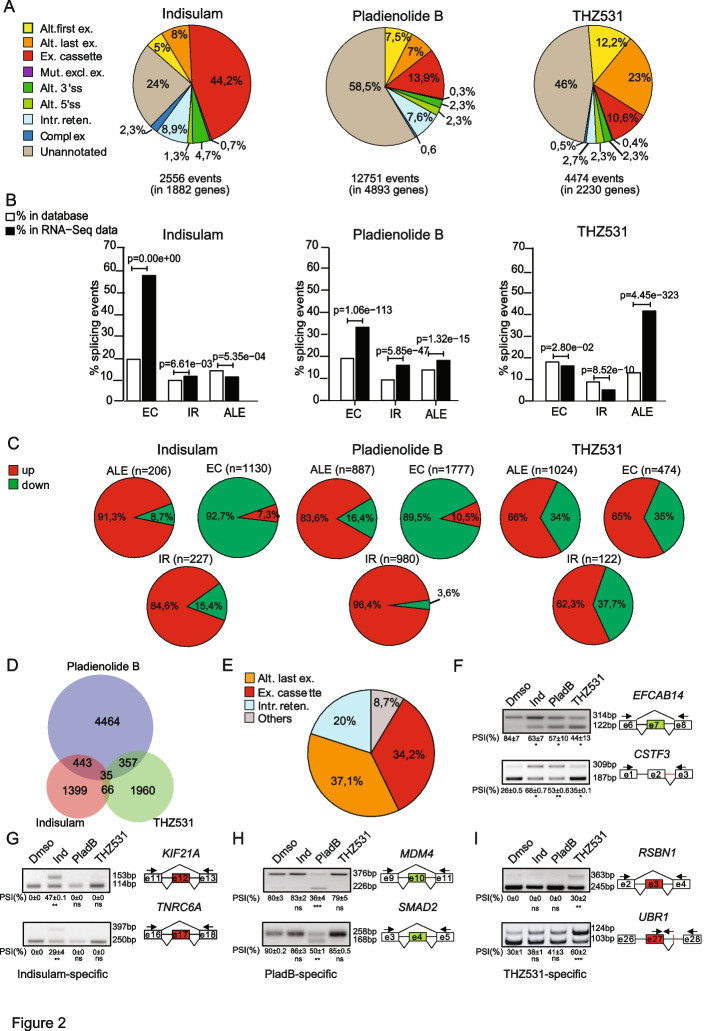
Indisulam, Pladienolide B and THZ531 modulate specific splicing patterns in 22Rv1 cells. **A** Pie chart showing % of events regulated in indicated splicing pattern upon indicated treatment in 22Rv1 (fold change ≥ 2, *p*-value ≤ 0.05). **B** Bar graphs showing the percentages of events for each splicing pattern annotated in FAST-DB (white columns) or regulated in 22Rv1 upon indicated treatment (black columns, modified Fisher’s test). **C** Pie charts showing percentages of up- (red) or down-regulated (green) events for indicated splicing pattern (ALE, alternative last exon; EC, exon cassette; IR, intron retention) in 22Rv1 upon indicated treatments.** D** Venn diagram showing the overlap between annotated splicing events regulated by the indicated in treatment in 22Rv1 compared to control cells. E) Pie chart representing distribution of 35 commonly regulated splicing events by the three treatments in 22Rv1 (see panel **D**) among different splicing patterns. **F**-**I** Representative images of RT-PCR analyses for overlapped (**F**), Indisulam—specific (**G**), PladB – specific (**H**) and THZ531- specific AS events in 22Rv1 cells treated or not for 24 h with indicated drugs (Ind, indisulam; PladB, pladienolide Schematic representation for each event is depicted beside the representative agarose gel. Red and green boxes indicate respectively up- and downregulated exons, black arrows indicate primers used for the PCR analysis. Percentage of spicing inclusion (PSI) was evaluated by densitometric analysis, and results are shown below agarose gels (mean ± SD, *n* = 3, t-test)

Inhibition of CDK12/13 was reported to impair the expression of genes with long introns [36, 37]. However, this feature was only associated with genes regulated at splicing level by THZ531 in 22Rv1 cells (Additional File 2: Fig. S2A). Indeed, the size of AS-only regulated genes is significantly larger than that of other non-regulated genes (*p* = 2,55^e−67^), and this feature is related to increased mean intron length (*p* = 4,95^e−63^; Additional File 2: Fig. S3A). The same structural organization was also observed for genes regulated at both GE and AS (GE/AS) level by THZ531 (Additional File 2: Fig. S2A, Additional File 2: Fig. S3A). Increased gene (*p* = 8,97^e−07^) and intron (*p* = 3,82^e−06^) length were also observed for genes regulated by indisulam at AS level, but not for those regulated by PladB (Additional File 2: Fig. S2A, Additional File 2: Fig. S3A). By contrast, THZ531 caused selective downregulation of small size genes (*p* = 6,65^e−24^), which are characterized by short introns and low intron/exon length ratio (Additional File 2: Fig. S2A, Additional File 2: Fig. S3A and B).

High transcriptional rate was postulated to enhance the sensitivity of cancer cells to splicing inhibition [7, 19]. In line with this concept, genes affected by indisulam (*p* = 4,58^e−04^) and PladB (*p* = 9,88^e−32^) at AS-only level are expressed at higher level than non-regulated genes in 22Rv1 cells, whereas this feature was not observed in genes regulated by THZ531 (Additional File 2: Fig. S2B). Collectively, these analyses highlight both common and unique features of the genes susceptible to regulation by different splicing inhibitors in CRPC cells.

### Pattern-specific effect of splicing targeting drugs

Exon cassette (EC) was the most regulated AS pattern in 22Rv1 cells treated with indisulam (44,2% of total events) and PladB (13,9% of total events), whereas THZ531 preferentially affected alternative last exon (ALE; 23%) events (Fig. 2A). THZ531 also affected a large fraction of alternative first exon (AFE; 12,2%) events, which are generally caused by selection of alternative promoters in the gene unit. We also found that EC and IR regulated events are significantly enriched in cells treated with indisulam and PladB with respect to their expected abundance in the FAST-DB reference database (Fig. 2B). Likewise, THZ531-modulated ALE events were also more abundant than expected (Fig. 2B). Thus, inhibition of the spliceosome mainly affects RNA processing events within the transcription unit of target genes, whereas inhibition of CDK12/13 preferentially alters last exon and polyadenylation site selection. Interestingly, IR and ALE events were preferentially upregulated by all drugs, whereas ECs were largely repressed by indisulam and PladB, but up-regulated by THZ531 (Fig. 2C).

Comparative analyses identified only 35 annotated events that were commonly regulated by the three drugs (Fig. 2D). Most of these common events were ALEs (37,1%), ECs (34,3%) or IRs (20%; Fig. 2E; Additional File 5: Supplementary Table S10). Functional validation of two of them by RT-PCR analyses (skipping of exon 7 in *EFCAB14* gene and retention of intron 2 in *CSTF3*) confirmed the bioinformatic prediction (Fig. 2F). However, the majority of the regulated AS events were specific for each treatment (Fig. 2D), indicating differential susceptibility of these exons and introns to the modality of splicing inhibition. RT-PCR analyses of arbitrarily selected events using independent 22Rv1 cell samples confirmed the drug-specific effects on splicing regulation (Fig. 2G-I).

### Splicing-targeting drugs deregulate exon cassette events with strong impact on CRPC cell survival

**Fig. 3:**
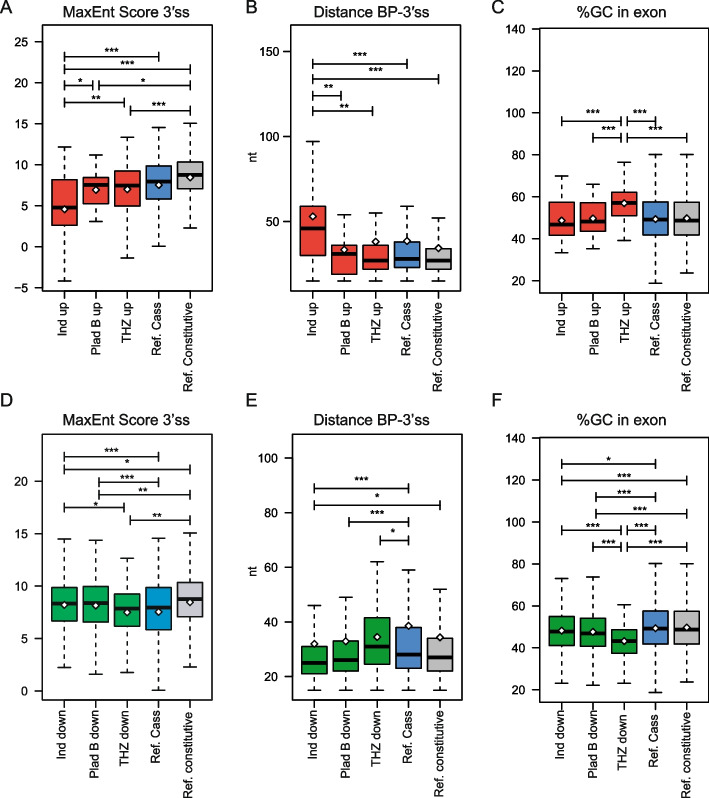
Specific sequence features characterize splicing-sensitive cassette exons to RNA splicing inhibitors in 22Rv1 cells. **A**-**F** Boxplots showing comparison between up-regulated exons (red boxes in **A**, **B**, **C**) or down-regulated exon (green boxes in **D**, **E**, **F**) by indicated splicing inhibitors (Ind = indisulam, PladB = Pladienolide B, THZ = THZ531) and other not-regulated cassette exons (ref. cassette, blue box) and constitutive exons (ref. constitutive, grey box) for strength of their 3' splice-site (**A**, **D**), distance of the branchpoint from the 3' splice-site (**B**, **E**) and % of GC content (**C**, **F**). Whiskers indicate 1.5 interquartile range and highlighted circles the mean values (*p ≤ 0.05, **p ≤ 0.01, ***p ≤ 0.001; ns = not significant, Welch’s t test). Number of analysed exons within each group is indicated in Additional File 2: FigureS5.

Indisulam and PladB deregulated > 1000 EC events in 22Rv1 cells. However, other ECs were unaffected by these treatments (Additional File 2: Fig. S4A). Thus, we sought to search for genomic features that confer sensitivity of exons to these spliceosome inhibitors. Indisulam and PladB inhibit, respectively, RBM39 and SF3B1, two factors involved in the recognition of the 3' ss by the U2 snRNP [7]. Surprisingly, exons up-regulated in the presence of indisulam are characterized by a 3' ss that is significantly weaker than that of non-regulated ECs (Fig. 3A). Moreover, these ECs displayed a larger distance between the 3' ss and the branchpoint (BP) sequence (Fig. 3B), a feature also known to diminish splicing efficiency [38]. These observations suggest that RBM39 represses weak cryptic exons that are not usually included in mature transcripts. Indeed, both visual inspection of the RNA-seq profiles and RT-PCR analyses indicated that indisulam-induced exons are almost not utilized in control 22Rv1 cells (Additional File 2: Fig. 5A and B). By contrast, exons repressed by both indisulam and PladB are characterized by a significantly stronger 3’ss that is more proximal to the BP (Fig. 3D,E). Moreover, these ECs are flanked by shorter introns with respect to other alternative exons (Additional File 2: Fig. S4B). These features were similar to those present in constitutive exons, suggesting that direct (PladB) and/or indirect (indisulam) weakening of U2 snRNP function preferentially impairs recognition of relatively strong exons. Interestingly, these features are not present in exons repressed by inhibition of CDK12/13. On the other hand, exons repressed by all three splicing targeting drugs, as well as their flanking introns, are characterized by a lower GC content, a feature that is more evident in THZ531-sensitive exons (Fig. 3F; Additional File 2: Fig. 5C). Moreover, THZ531-upregulated ECs are characterized by high GC content (Fig. 3C). Thus, the GC content of an exon makes it particularly sensitive to the RNAPII phosphorylation status. Other features of the regulated exons, such as the 5’ss, the BP and the polypyrimidine tract strength, were less relevant for the sensitivity of ECs to these inhibitors (Additional File 2: Fig. S4D E and F).

**Fig. 4:**
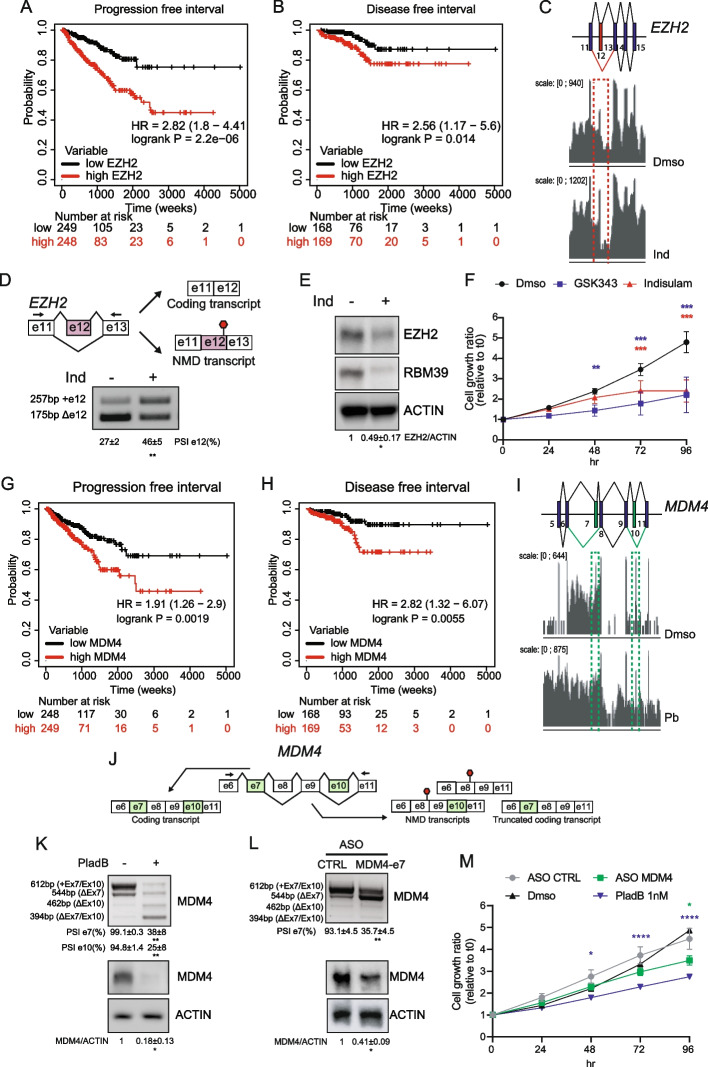
Alternative cassette exons in *EZH2* and *MDM4* genes affects 22Rv1 cells viability. **A**, **B** Kaplan Meier curves illustrating the progression (**A**) and disease free interval (**B**) of prostate adenocarcinoma (PC) patients of the TCGA cohort, segregated according to median expression levels of the *EZH2* gene. **C** Schematic representation and profile of the RNA-seq reads of the indicated region of *EZH2* gene in 22Rv1 cells treated with Indisulam (Ind) or Dmso. Sequence reads (vertical gray lines), exons (blue boxes), and introns (horizontal lines) are shown. Dashed red box highlights the RNA-seq reads along the upregulated exon 12 (red box). **D** Schematic representation of *EZH2* exon 12 alternative cassette exon. Black arrows indicate primers used for the RT-PCR analyses of its inclusion in 22Rv1 treated or not with Ind (3,3 µM, 12 h). Percentage of spicing inclusion (PSI) is shown below the representative agarose gel (densitometric analysis, mean ± SD, *n* = 3, one-way Anova). NMD, non-sense mediated decay. **E** Representative Western Blot analysis for EZH2 protein levels in 22Rv1 cells treated or not for 24 h with Ind (3,3 µM). ACTIN was evaluated as loading control. Densitometric analysis of the EZH2/ACTIN ratio is shown below the blots (mean ± SD, *n* = 4, t-test). **F** Line graph showing growth rate of 22Rv1 cells treated with the EZH2 inhibitor GSK343 (10 µM) and Indisulam (3,3 µM), evaluated as cell confluence ratio relative to time 0 (t0; mean ± SD, *n* = 4, two-way Anova). G, H) Kaplan Meier curves illustrating the progression (**G**) and disease (**H**) free interval of PC patients of the TCGA cohort, segregated according to median expression levels of the *MDM4* gene. **I** Schematic representation and profile of the RNA-seq reads of the indicated region of *MDM4* gene in 22Rv1 cells treated with Pladienolide B (PladB) or Dmso. Sequence reads (vertical gray lines), exons (blue boxes), and introns (horizontal lines) are shown. Dashed green box highlights the RNA-seq reads along the downregulated exons 7 and 10 (green boxes). **J** Schematic representation of the alternative cassette exon events involving exon 7 and 10 of *MDM4* gene. Black arrows indicate primers used for the RT-PCR analyses. **K**, **L** RT-PCR analysis of *MDM4* exons 7 and 10 inclusion (upper panels) and western blot analysis (lower panels) of MDM4 protein levels in 22Rv1 treated or not with Plad B (10 nM, 6 h) (**K**) and in 22Rv1 transduced with control or MDM4 exon 7 targeting antisense oligonucleotide (ASO). ACTIN was evaluated as loading control. Results of the densitometric analysis of the PSI are shown below the agarose gel (mean ± SD, *n* = 3, t-test). Densitometric analysis of the MDM4/ACTIN ratio is shown below the blots (mean ± SD, *n* = 3, t-test). M) Line graph showing growth rate of 22Rv1 cells transduced with ASO-CTRL or ASO-MDM4 or treated with PladB (1 nM) or Dmso, evaluated as cell confluence ratio relative to t0 (mean ± SD, *n* = 2, two-way Anova)

Since splicing-targeting drugs strongly affect the viability of CRPC cells, we searched for specific splicing changes that could affect cell survival. To this end, we focused on ECs whose inclusion/skipping may alter the expression of genes that exhibit a direct relevance for CRPC. Indisulam specifically regulates the AS of EZH2 (Additional File 3: Table S3-S4), a gene associated with CRPC malignancy and whose inhibition enhances sensitivity to genotoxic stress [39, 40]. In line with its oncogenic function, clinical data in The Cancer Genome Atlas (TCGA) indicated that high expression of EZH2 correlates with shorter progression-free (PFI) and disease-free interval (DFI) in PC patients (Fig. 4A and B). RNA-seq analysis indicated that indisulam induces the selective inclusion of exon 12 in *EZH2* (Fig. 4C). This splicing event introduces a premature stop codon that was shown to target the transcript for degradation by the non-sense mediated decay (NMD) pathway (Fig. 4D) [41]. We confirmed the specific effect of indisulam on the inclusion of *EZH2* exon 12 with respect to PladB and THZ531 (Fig. 4D, Additional File 2: Fig. S5C). Moreover, in line with the expected NMD-linked effects, indisulam reduced EZH2 protein expression in 22Rv1 cells (Fig. 4E). This effect is functionally relevant for CRPC cells, as treatment with indisulam mimicked the effect of the EZH2-specific inhibitor GSK343 on the inhibition of cell proliferation (Fig. 4F) and on the reduction of H3K27me3 levels at 72–96 h of treatment (Additional File 2: Fig. S5D). Another target with direct relevance for CRPC is *MDM4*, a gene recently implicated in predicting progression of PC to an advanced stage [42]. Analysis of TCGA data indicated that high MDM4 expression is associated with shorter PFI and DFI in PC patients (Fig. 4G,H). PladB induced skipping of exon 7 and exon 10 in this gene (Fig. 4I). Notably, exon 7 skipping was shown to target the MDM4 transcript to NMD (Fig. 4J) and to impair MDM4 protein expression in other cell types [43]. RT-PCR analysis validated the bioinformatic prediction, showing that PladB treatment causes skipping of both exon 7 and exon 10 (Fig. 4K, Additional File 2: Fig. S5F). In line with the prediction that exon 7 skipping targets the MDM4 transcript to NMD, PladB treatment strongly reduced MDM4 protein expression in 22Rv1 cells (Fig. 4K). To test whether MDM4 inhibition by this splicing event was sufficient to impair CRPC cell viability, we developed an antisense oligonucleotide (ASO) targeting the 3’ ss of exon 7. Transfection of the ASO was sufficient to induce exon 7 skipping and MDM4 protein depletion (Fig. 4L). More importantly, selective switch of MDM4 exon 7 splicing was sufficient to significantly reduced viability of 22Rv1 cells (Fig. 4M), thus recapitulating the effect exerted by PladB. These findings indicate that splicing dysregulation of specific genes associated with malignancy has a direct impact on CRPC cell viability.

### Splicing targeting drugs prevalently impair intron excision in CRPC cells

**Fig. 5:**
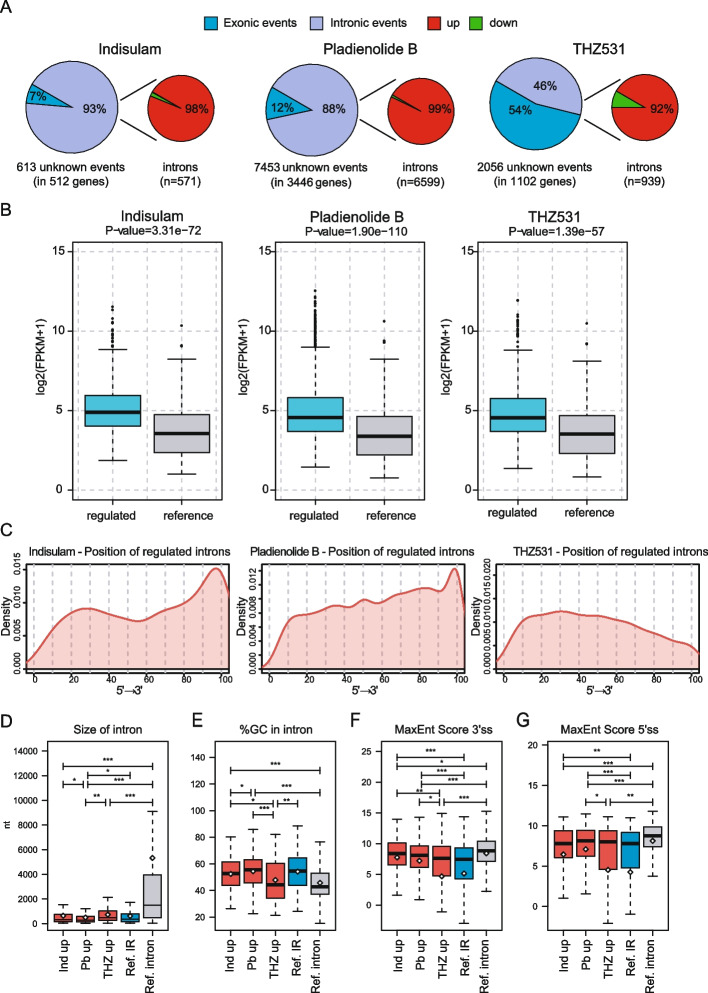
Pervasive intron retention is induced by splicing inhibitors in 22Rv1 cells. **A** For each indicated drug, the left pie chart illustrates the percentages of exonic (light blue) and intronic (purple) regulated events and the right pie chart highlights the percentages of up- (red) and downregulated (green) events within the subset of intronic events. **B** Box plots showing the expression levels (log2 FPKM) of regulated (light blue bar) and unaffected genes (reference, grey bar) by intron-retention in 22Rv1 cells treated with indicated drugs. **C** Curve graphs showing distribution of the upregulated introns (red line) by indicated drugs within the transcription unit of their hosting genes (5′ → 3’ direction). **D**-**G** Boxplots showing comparison between up-regulated introns (red boxes) by indicated splicing inhibitors (Ind = indisulam, Pb = Pladienolide B, THZ = THZ531) and other not-regulated intron-retention events (ref. intron, blue boxes) and properly spliced-introns (ref. constitutive, grey box) for indicated features. In B and D-G Whiskers indicate 1.5 interquartile range and highlighted circles the mean values (Welch’s t test). Number of analysed exons within each groups is indicated in Additional File 2: Fig S7.

A large percentage of the AS events altered by the splicing targeting drugs are unannotated in the FAST-DB database (Fig. 2A). Most of these events correspond to retained introns (92–99%; Fig. 5A), indicating that all three inhibitors cause a widespread impairment of the splicing machinery in CRPC cells. Although there is a very limited overlap between the introns regulated by these drugs (Additional File 2: Fig. S6A and B), all IR events were significantly enriched within genes that are highly expressed in 22Rv1 cells (Fig. 5B).

To further investigate the nature of introns that are sensitive to drug-induced splicing inhibition, we analysed their structural features. Introns that are up-regulated by indisulam and PladB accumulate toward the 3’ end of genes (Fig. 5C), as shown for other physiological or transcription-regulated IR events [29, 44]. By contrast, as reported in other cell types [34, 45] inhibition of CDK12/13 activity prevalently induced IR in the proximal region of the transcriptional unit (Fig. 5C). Furthermore, all three drugs caused retention of introns that are significantly shorter than constitutive introns (Fig. 5D), in line with the notion that regulated introns display exon-like features, with shorter size and higher GC content in their sequence [29, 44]. However, PladB-retained introns are particularly short, with significantly reduced size compared to those regulated by indisulam or THZ531, as well as to other annotated IR events that are not regulated by any of these drugs in 22Rv1 cells (Ref. IR; Fig. 5D). Moreover, while introns up-regulated by PladB and indisulam display the typical high GC content observed in retained introns, the GC content of introns susceptible to CDK12/13 inhibition is comparable to that of constitutively spliced introns (Fig. 5E). THZ531-regulated introns also differed with respect to splice site features. While both spliceosome inhibitors impaired splicing of introns featuring 5’ and 3’ splice sites that are stronger than those of other non-regulated IR events, THZ531-regulated introns were indistinguishable from them (Fig. 5F and G). On the other hand, introns regulated by all three drugs were characterized by stronger BP sequences with respect to other reference IR events and constitutive introns (Additional File 2: Fig. S6C). These data indicate that pharmacologic inhibition of the spliceosome mainly affects processing of “exon-like” small introns characterized by a high GC content, whereas inhibition of CDK12/13 impairs splicing of introns characterized by weaker splice sites and lower GC content.

### Inducible intron retention and alternative last exon selection affect the expression of CRPC-relevant genes

Given the pervasive nature of IR in 22Rv1 cells treated with splicing-targeting drugs, we searched for intronic splicing events that may elicit functional consequences in CRPC. First, we noticed that the intronic region between exon 1 and exon 3 of the androgen receptor gene (*AR*) was significantly up-regulated by treatment with THZ531 (Additional File 2: Fig. S7A,B). This event was associated with strong repression of the expression of downstream regions of the AR transcript (Additional File 2: Fig. S7C) and with significant reduction of AR protein expression, including the ARv7 truncated variant that confers androgen-resistance to CRPC (Additional File 2: Fig. S7F, G and H). A similar reduction of AR transcript and protein levels, including that of the ARv7 isoform, was also observed upon treatment with PladB (Additional File 2: Fig S7D, S7E and S7I-K), which caused retention of several introns in the *AR* gene (Additional File 3: Tables S5 and 6). Another clinically relevant target is *ERBB3*, which encodes for an epidermal growth factor (EGF) tyrosine kinase receptor. ERBB3 promotes PC cell survival upon androgen deprivation therapy and was proposed as an actionable target in advanced PC [46, 47]. Treatment with all three splicing inhibitors induced selection of an alternative polyadenylation (APA) site in *ERBB3* intron 5 and premature termination of the transcript at this ALE (Additional File 2: Fig. S7L). This effect was likely due to intron 5 retention, as visual inspection showed increased read coverage also downstream of the selected intronic polyadenylation site (Additional File 2: Fig. S7L). RT-PCR and Western blot analyses confirmed selection of this ALE and repression of the full-length ERBB3 transcript and protein by the three drugs (Additional File 2: Fig. S7M-R), thus confirming the functional relevance of this splicing defect.

**Fig. 6:**
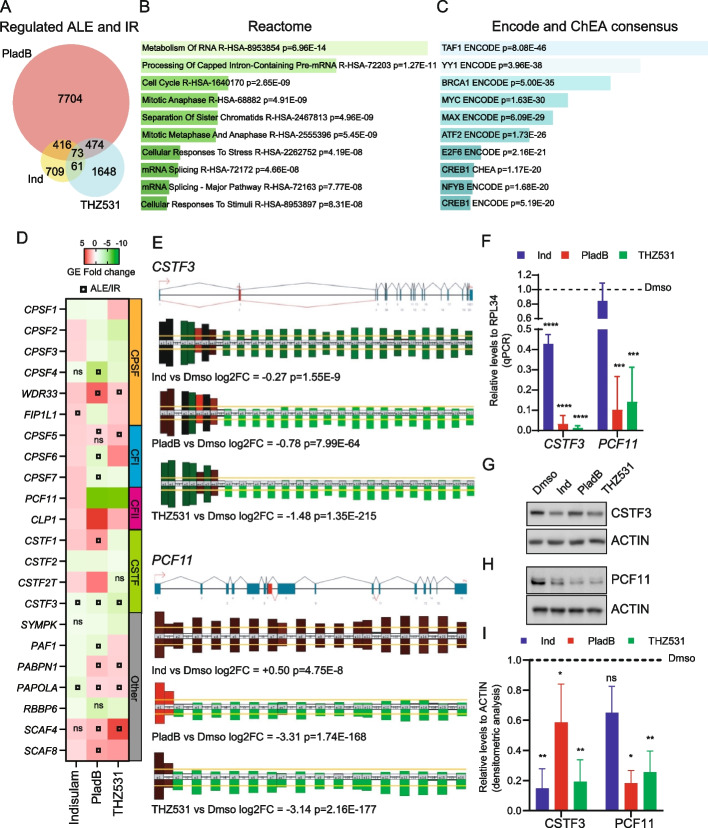
Splicing targeting drugs affects 3’-end processing genes through intron retention and alternative last exon selection. **A** Venn diagram showing the overlap of regulated alternative last exon (ALE) and intron-retention (IR) events regulated by Indisulam (Ind), Pladienolide B (PladB) and THZ531 in 22Rv1 cells, according to our RNA-seq data. **B**, **C** Bar graph illustrating for genes regulated by IR and ALE by at least two drugs (see panel A) annotation of significantly enriched pathways in the Reactome database (**B**) and putatively regulatory transcription factors according to query of indicated ChiP-seq databases (**C**) (analysis performed with Enrichr tool, *p*-value < 0.05). **D** Heatmap showing the gene-expression fold-change for indicated genes of the cleavage and polyadenylation (CPA) machinery of 3’-end mRNA: CPA specificity factor (CPSF), cleavage factor I (CFI) and II (CFII), cleavage stimulation factor (CSTF) and other genes according to RNA-seq data of 22Rv1 treated with indicated drugs with respect to DMSO treated cells. Circles inscribed in a square indicate regulation by an ALE or IR event. **E** Schematic visualization in the EASANA database of the RNA-seq reads profile for *CSTF3* (upper panel) and *PCF11* (lower panel) genes in 22Rv1 cells treated with either Ind, Plad B, THZ531 with respect to the DMSO. *P*-value and log2 of the gene expression fold-change with respect to DMSO are indicated. F) qPCR analysis of *CSTF3* and *PCF11* full length transcripts in 22Rv1 cells treated (for 16 h) with indicated drugs compared to control. RPL34 was evaluated as loading control (mean ± SD, *n* = 3 one-way Anova). **G**, **H** Representative Western Blot analysis for CSTF3 (**G**) and PCF11 (**H**) in 22Rv1 cells treated for 24 h (**G**) or 16 h (**H**) with indicated drugs. ACTIN was evaluated as loading control. I) Bar graph showing the results of the densitometric analysis of the ratio of expression of indicated proteins with respect to ACTIN (mean ± SD, *n* = 3, one-way Anova)

Next, we searched for pathways that are particularly affected by IR and/or internal ALE selection in CRPC cells. Genes regulated by IR/ALE upon treatment with at least two of the drugs used in our study (*n* = 1024; Fig. 6A) were significantly enriched in terms related to RNA metabolism and RNA processing regulation (Fig. 6B). IR/ALE-regulated genes were also enriched for targets of MYC (Fig. 6C), which is known to dysregulate RNA processing regulation in PC [24]. Notably, several studies have recently shown that widespread alteration of 3’-end RNA processing in PC cells contributes to tumour progression [48–51]. Moreover, androgen deprivation therapy in PC cells induced a general reprogramming of APA, by promoting the pattern observed in CRPC cells [48]. These observations suggest that APA dysregulation contributes to PC progression and to acquisition of a therapy-resistant phenotype. Thus, we focused on this pathway for further analysis. The 3’-end processing of nascent transcripts requires the cleavage and polyadenylation specificity factor (CPSF) complex, which binds to the polyadenylation signal (PAS). In addition, the cleavage stimulation factor (CSTF) and the cleavage factor I (CFI) and II (CFII) complexes recognize sequence elements flanking the PAS and are required for definition of the cleavage site [52]. First, we interrogated the RNA-seq data for changes at GE or ALE/IR level of the genes encoding for the protein components of these four complexes. All these genes were significantly modulated at GE level by at least two of the splicing inhibitors tested, with PladB and THZ531 causing significant dysregulation of > 90% of the genes required for 3’-end RNA processing (Fig. 6D). Moreover, several genes were also affected at IR/ALE level upon treatment with PladB or THZ531 (Fig. 6D). For instance, both drugs caused selection of an upstream ALE in the *WDR33* gene (Additional File 2: Fig. S8A), encoding for the CPSF factor that binds the PAS sequence [52]. The same ALE was also upregulated by indisulam, albeit it did not reach statistical significance in our analysis (Supplementary Fig. S8A). Consistently, RT-PCR analysis showed an increased retention of intron 6 in 22Rv1 cells treated with all splicing inhibitors, and a concomitant reduction of the full-length transcript (Additional File 2: Fig. S8B-D). Likewise, all three drugs caused usage of an ALE in *CSTF3* intron 3 (Fig. 2F; Fig. 6E), with consequent reduction of the downstream reads and of the expression of the full-length CSTF3 transcript (Fig. 6F). A similar regulation was also observed for *PCF11*. Indeed, although our analysis indicated that PCF11 expression was downregulated by PladB and THZ531 (Fig. 6D), this effect was associated with a strong retention of intron 1 and premature termination of the transcript within this intron (Fig. 6E), a type of regulation previously described in other cell contexts [53, 54]. Also in this case, we confirmed that both drugs caused a downregulation of PCF11 mRNA expression (Fig. 6F). In addition, Western blot analyses showed significant downregulation of CSTF3 and PCF11 protein levels by PladB and THZ531 (Fig. 6G-I). Collectively these observations show that the 3’-end RNA processing pathway is highly sensitive to splicing inhibition in CRPC cells.

**Fig. 7:**
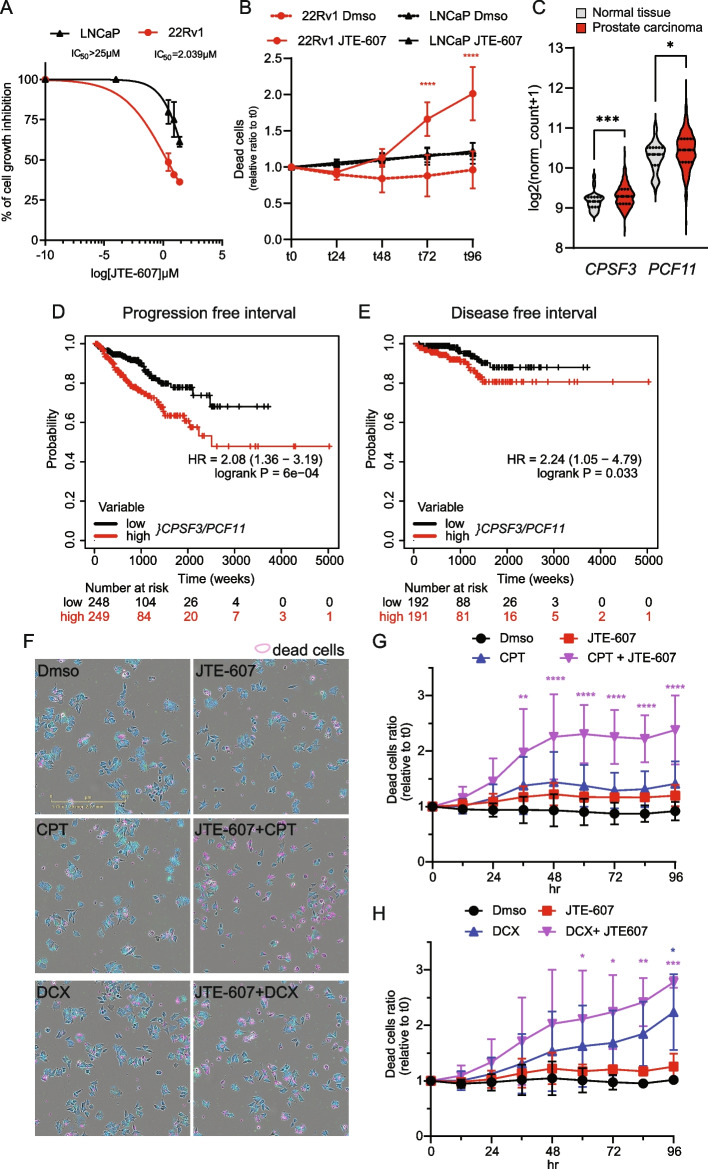
JTE-607 inhibitor exerts anti-tumoral activity in CRPC cells. **A** Dose–response curve illustrating the growth inhibitory effects of JTE-607 treatment on 22Rv1 cells. Half maximal inhibitory concentration (IC_50_) for the two cell lines is indicated (mean ± SD, *n* = 2). **B** Line graph showing the cell death ratio of LNCaP and 22Rv1 cells treated with 25 µM JTE-607or Dmso at indicated time point (mean ± SD, *n* = 3, two-way Anova). **C** Violin plot showing the expression levels of *CPSF3* and *PCF11* genes in primary prostate adenocarcinoma (PC, *n* = 429) or normal prostate tissue (*n* = 43) (TCGA dataset, median value is highlighted, Welch Anova test). **D**, **E** Kaplan Meier graphs of the progression free interval (D) and disease free interval (**E**) survival probability of PC patients in the TCGA database classified according to the expression levels of *CSTF3/PCF11* genes. **F** Representative image of Incucyte® Cytotox green stained 22Rv1 at 48 h of treatment with indicated drugs (JTE-607 6 µM; CPT, cisplatin 1 µM, DCX, docetaxel 3 nM). Violet boundaries highlight cells positive for cytotox green dye. Nuclight Rapid NIR (blue color) stains cells nuclei. **G**, **H** Line graphs showing either the cell death ratio of 22Rv1 treated with either 6uM JTE-607 and CPT 1 µM (**G**, mean ± SD *n* = 3) or DCX (3 nM) (H, mean ± SD *n* = 2) alone or in combination with respect to vehicle (Dmso) treated cells (two-way Anova)

Since alterations in this pathway have been linked to PC development and acquisition of a castration resistant status [48–50], we aimed at testing the impact of its pharmacologic inhibition on CRPC cell viability. A specific inhibitor of CPSF3, the CPSF catalytic component that operates the cleavage at the polyadenylation site [52] was recently developed [55]. This drug, named JTE-607, displayed high efficacy in selected tumors, such as acute myeloid leukemia and Ewing’s sarcoma [56]. More recently, it was proposed that JTE-607 specificity as anticancer agent relies on high 3’-end processing activity of the cell [57]. Interestingly, 22Rv1 cells were highly sensitive to JTE-607, with IC50 < 5 µM (Fig. 7A), similarly to what reported for tumour cells that are vulnerable to this drug [56, 57]. By contrast, LNCaP, which are representative of a less aggressive and androgen-sensitive stage of PC [48], were much less sensitive (IC_50_ > 25 µM; Fig. 7A, Additional File 2: Fig. 8E). Moreover, 22Rv1 cells, but not LNCaP, underwent cell death upon treatment with JTE-607 (Fig. 7B). JTE-607-sensitive genes were recently proposed to share features with those affected by PCF11 knockdown [57]. Interestingly, knockdown of PCF11 also caused a significant reduction of 22Rv1 cells proliferation (Additional File 2: Fig. S8F,G), partly mimicking the effect of CPSF3 inhibition. Since CPSF3 expression is also negatively affected by both PladB and THZ531 treatment (Fig. 6D, Additional File 2: Fig. S8H,I), these findings suggest that 22Rv1 cells particularly rely on these two 3’-end processing factors and that their repression could contribute to the anti-cancer activity of splicing-targeting drugs.

Transcriptomic data from TCGA revealed that expression of both PCF11 and CPSF3 genes is elevated in PC patients with respect to normal prostate tissue (Fig. 7C). CPSF3 expression was shown to be higher in PC patients that experienced disease relapse [48]. Moreover, high expression of CPSF3 and PCF11 was significantly associated with shorter PFI and DFI in PC patients (Additional File 2: Fig. S8J-K), while their concomitant up-regulation was an even more significant indicator of poor prognosis (Fig. 7D,E). Then, since treatment of advanced PC relies on cytotoxic chemotherapy, we asked whether inhibition of the 3’-end processing pathway affected the response to cisplatin and docetaxel. Importantly, treatment of 22Rv1 cells with sub-optimal doses of JTE-607 increased the cytotoxic effects elicited by cisplatin and docetaxel, two agents used in the clinic for this cancer [21], (Fig. 7F,G). These results suggest that the 3’-end processing pathway is an actionable vulnerability in advanced PC, whose inhibition synergizes with current chemotherapeutic treatments for the disease.

## Discussion

Prostate carcinogenesis and insurgence of the CR status are promoted by aberrant expression of the oncogene MYC and widespread deregulation of AS [24, 25]. At the same time, AS deregulation increases the sensitivity of CRPC, as well as several other tumour types, to the anti-tumoral activity of inhibitors of the splicing process and of transcriptional kinases, such as CDK12/13 [7, 12, 19, 25]. In this study, we elucidated the global transcriptome changes underlying the strong anti-tumoral effects elicited by treatment with indisulam, PladB and THZ531 in 22Rv1 cells, an established model of CRPC. In spite of the similar effects exerted on inhibition of cell proliferation and induction of cell death, each drug altered the transcriptome of 22Rv1 cells in a specific manner. Indisulam modulated the expression of a smaller number of genes with respect to PladB and THZ531 (4% versus > 25% of the expressed genes), and it preferentially repressed gene expression (> 65% of the regulated genes). Moreover, we observed a smaller overlap between genes sensitive at GE and AS level in indisulam-treated cells. This result suggests that indisulam elicits GE changes by a selective effect on specific promoters, rather than by causing indirect changes in RNA stability through AS changes. This notion is in line with previous studies indicating a direct role for RBM39 as transcriptional co-factor (62). Gene length is another discriminant feature for drug-specific susceptibility. Indeed, the expression of intron-less or intron-poor genes is specifically induced only by treatment with indisulam and PladB. Upregulation of short genes by drugs that directly inhibit splicing factors may represents a rapid stress response of cancer cells, which react to the reduced splicing efficiency by enhancing the expression of genes that do not rely on this process. Interestingly, it was previously shown that human intronless genes are enriched for signaling and regulatory molecules that are relevant for cell growth and proliferation [58] These genes may have evolved to withstand harsh conditions of limited energy availability, and global inhibition of splicing may mimic such condition.

Indisulam, PladB and THZ531 co-regulated a common subset of 476 genes at AS level, which were enriched in functional categories of direct relevance for PC. However, the number of common splicing events was quite small (*n* = 35), suggesting the existence of genes that are prone to AS regulation, albeit at different exons/introns and through different regulatory mechanisms. Interestingly, recent evidence suggests that splicing efficiency is dictated by specific structures and three-dimensional organization of the chromatin [59–61]. It is possible that these co-regulated genes that are particularly susceptible to splicing perturbations reside in the same chromatin territories within the nucleus. Genes regulated at splicing level by indisulam and PladB are also expressed at significantly higher levels in 22Rv1 cells. This result is in line with the enhanced sensitivity to splicing inhibition of cells displaying high transcriptional rate [12]. Thus, an increase in transcription is tolerated by the cell, or by an individual gene, only if a highly efficient splicing machinery is available. Our observation is also in line with the hypothesis that competition between nascent transcripts for the spliceosome regulates splicing outcome [62], especially in a condition of reduced splicing efficiency. Regarding the effect of THZ531, a correlation between high expression and defective splicing was only observed for IR-regulated genes. Moreover, CDK12/13 inhibition particularly affected long genes in 22Rv1 cells. These findings suggest that these kinases ensure an efficient coupling between transcription and splicing, which may be particularly relevant for large transcription units and for the productive processing of long introns.

IR, EC and ALE were the predominant splicing patterns regulated by the three drugs, beyond their expected frequency in the reference database. However, while IR and ALE events were induced by all three drugs, a different trend was observed for EC regulation. Indisulam and PladB promoted skipping of most of the regulated ECs, coherently with their inhibitory activity towards U2 snRNP-related functions [7]. By contrast, THZ531 treatment preferentially induced their inclusion. Importantly, ECs promoted by THZ531 feature a weak 3’ ss, suggesting that their recognition by the spliceosome may benefit of a reduced transcriptional elongation rate. This hypothesis is in line with the kinetic model of splicing regulation, which suggest that a slow polymerase creates a window of opportunity for weak ss before stronger ones in distal exons are transcribed and compete them out [3, 4]. On the other hand, ECs repressed by indisulam and PladB are characterized by a stronger 3' ss with respect to other unregulated ECs. This unexpected finding suggests that a reduced efficiency/fidelity of the U2 snRNP might unleash the repressive activity of negative splicing regulators.

Next, we asked whether specific AS events may contribute to the anti-tumoral activity of splicing-targeting drugs. To address this question, we focused on two model genes: *EZH2* and *MDM4*. Importantly, high expression of both genes was significantly associated with poor prognosis in PC. Our work now shows that indisulam and PladB promote splicing events that directly impair their expression. Moreover, the reduced expression/function of EZH2 and MDM4 proteins may contribute to the anti-tumoral activity of these drugs, as their pharmacologic (EZH2) or RNA-mediated (MDM4) inhibition largely recapitulated the effects of indisulam and PladB on 22Rv1 proliferation. Thus, the AS events identified by our transcriptomic analyses represent a valuable repertoire of putative therapeutic targets, which can be mined to identify new actionable vulnerabilities for CRPC and, possibly, other cancers. In this regard, the uprising development of highly specific RNA technologies might accelerate the generation of tools that efficiently modulate splicing events also in human cancers, like those already available for other diseases [6, 7, 63].

IR is the most prominent splicing pattern altered by pharmacologic inhibition of the spliceosome or CDK12/13 in 22Rv1 cells. Augmented IR was proposed to open multiple therapeutic windows for cancer. On one hand, translation of intron-retaining transcripts induced by splicing inhibitors can generate neoepitopes and induce tumor-selective immune responses [18, 64]. Although this aspect was not addressed herein, our transcriptomic dataset represents an explorable catalogue of actionable introns, which can be investigated for their immunogenic potential. On the other hand, IR events induced by CDK12/13 inhibition were shown to enhance vulnerability of cancer cells to chemotherapeutic treatments. For instance, pharmacologic inhibition of these kinases reduced the expression of genes involved in the DNA damage response (DDR) and to enhance the sensitivity of breast and ovarian cancer cells to PARP (PARPi) and other DDR inhibitors [36, 37, 45]. Herein, we show that THZ531 induces retention if the first intron in the *AR* gene, causing a strong reduction of both its full-length and oncogenic splice variants. A similar effect on *AR* expression was also elicited by PladB, which caused retention of several downstream introns in the gene. Moreover, all three splicing targeting drugs caused IR and downregulation of ERBB3 expression, a growth factor receptor associated with PC progression (49, 50).

Analyses of IR-regulated genes also uncovered the strong dependency of 22Rv1 cells on proper regulation of 3’-end mRNA processing. CPA genes were enriched among the genes regulated by IR/ALE and at GE level by splicing inhibitors. In some cases, like *CSTF3* and *PCF11*, the IR event negatively affected expression of the full-length transcript and protein. These observations suggested that impaired functionality of the CPA machinery could contribute to the cytotoxic effects of splicing-targeting drugs. This hypothesis is supported by our observation that 22Rv1 cells are highly sensitive to JTE-607, a catalytic inhibitor of CPSF3. Notably, CPSF3 expression is negatively affected by PladB and THZ531. Moreover, knockdown of PCF11, another target of both drugs, was shown to phenocopy the effects of JTE-607 treatment [57], and treatment with JTE-607 or knockdown of PCF11 reduced proliferation of 22Rv1 cells. JTE-607 significantly induced cell death in 22Rv1, but not of the less aggressive LNCaP cells. Thus, since sensitivity to CPSF3 inhibition was associated with increased 3’-end processing activity of the cell [57], our data suggest that this process correlates with a more advanced stage of disease. Importantly, our study also shows that treatment with suboptimal doses of JTE-607 can sensitize 22Rv1 cells to the cytotoxic effects of chemotherapeutic agents used for advanced CRPC, such as cisplatin and docetaxel. Together with the observation that concomitant high levels of CPSF3 and PCF11 represent a negative prognostic factor for PC patients, these data strongly suggest that pharmacological inhibition of the CPA process represents a valuable therapeutic opportunity for advanced CRPC.

## Conclusions

Our study highlights the cis-acting regulatory features underlying susceptibility to transcriptional and post-transcriptional variations induced by splicing-targeting drugs; it reveals IR as the prominent pattern induced by both direct (Ind/PlaB) and indirect (THZ531) inhibition of splicing and uncovers the 3’-end mRNA processing as a druggable vulnerability for advanced PC.

### Supplementary Information

**Additional file 1:** **Supplementary Table 1.** List of drugs used in the study, **Supplementary Table 2.** List of primers used in this study.

**Additional file 2.** Supplementary Figures and Figure legends.

**Additional file 3:** **Supplementary Table S3.** List of differentially expressed genes in Indisulam treated 22Rv1 cells compared to control (fold change >=2, *p*-value<=0.05), **Supplementary Table S4.** List of differentially spliced events in Indisulam treated 22Rv1 cells compared to control (fold change >=2, *p*-value<=0.05), **Supplementary Table S5.** List of differentially expressed genes in Pladienolide B treated 22Rv1 cells compared to control (fold change >=2, *p*-value<=0.05), **Supplementary Table S6.** List of differentially spliced events in Pliadenolide B treated 22Rv1 cells compared to control (fold change >=2, *p*-value<=0.05), **Supplementary Table S7.** List of differentially expressed genes in THZ531 treated 22Rv1 cells compared to control (fold change >=2, *p*-value<=0.05), **Supplementary Table S8.** List of differentially spliced events in THZ531 treated 22Rv1 cells compared to control (fold change >=2, *p*-value<=0.05).

**Additional file 4:** **Supplementary Table S9.**

**Additional file 5:** **Supplementary Table S10.** List of annotated splicing events regulated by Pladienolide B, Indisulam and THZ531 in 22Rv1 cells compared to control (fold change >=2, *p*-value<=0.05).

## Data Availability

The RNA-seq data underlying this article are available in Gene Expression Omnibus repository at https://www.ncbi.nlm.nih.gov/geo/ and can be accessed with GSE234734.

## Abbreviations

PC: Prostate cancer
CR: Castration-resistance
CRPC: Castration-resistant prostate cancer
AS: Alternative splicing
GE: Gene expression
PladB: Pladienolide B
Ind: Indisulam
RNAPII: RNA polymerase II
Ss: Splice site
BP: Branchpoint
IR: Intron retention
EC: Exon cassette
ALE: Alternative last exon
AFE: Alternative first exon
TCGA: The Cancer Genome Atlas
PFI: Progression-free interval
DFI: Disease-free interval
ASO: Antisense oligonucleotide
CPSF: Cleavage and polyadenylation specificity factor
DDR: DNA damage response
