# Supplementary material for: Splicing targeting drugs highlight intron retention as an actionable vulnerability in advanced prostate cancer
Source: J Exp Clin Cancer Res. 2024 Feb 27;43:58. doi: 10.1186/s13046-024-02986-0 (PMC10898177; doi:10.1186/s13046-024-02986-0)
Supplement: Supplementary file 2 — Additional file 2. Supplementary Figures and Figure legends. [file 13046_2024_2986_MOESM2_ESM.pdf]

Additional File 2: Supplementary Figures and Figure legends

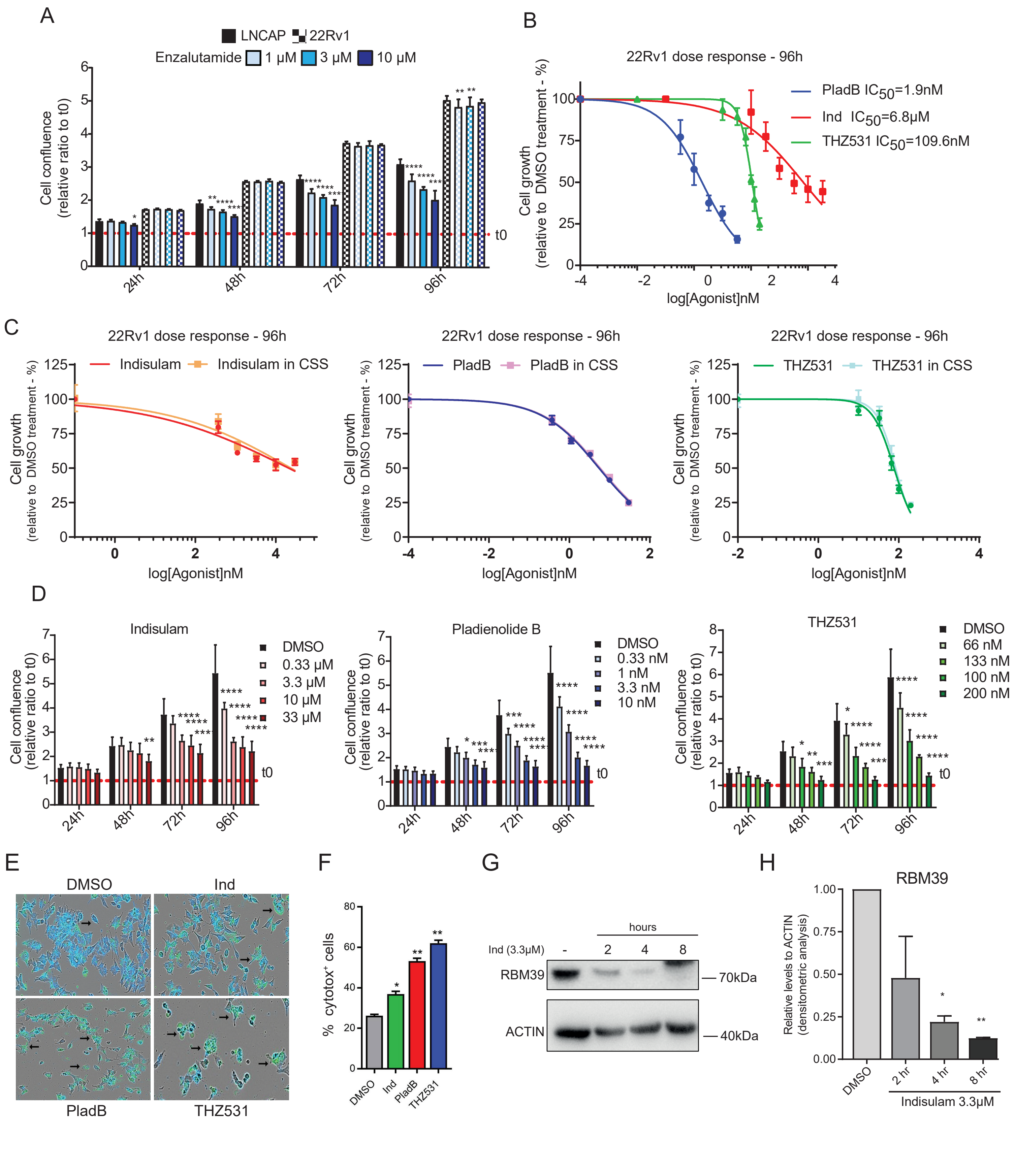

Supplementary Figure S1

Supplementary Figure S1. Castration-resistant 22Rv1 cell line is sensitive to splicing-targeting drugs. A) Bar graph showing the growth rate of androgen-sensitive LNCaP (solid bars) and castration resistant 22Rv1 (barred bar) cell lines, treated with indicated doses of enzalutamide, evaluated as cell confluence ratio relative to time 0 (set to 1 and indicated by a red dashed line, mean± SD, n=3, two-way Anova). B) Dose-response curves illustrating the growth inhibitory effects of the splicing targeting drugs Indisulam (Ind), Pladienolide B (PladB) and THZ531 on 22Rv1 cells. Cell growth was evaluated as measure of the cell confluence after 96h of treatment with respect to vehicle (DMSO) treated cells, set to 100%. Half maximal inhibitory concentration (IC50) for each drug is indicated (mean± SD, n=3). C) Dose-response curves illustrating the growth inhibitory effects of the three indicated splicing targeting drugs on 22Rv1 cells grown in media supplemented with 10% charcoal-stripped serum (CSS). Cell growth was evaluated as measure of the cell confluence after 96h of treatment with respect to vehicle (DMSO) treated cells, set to 100%. Half maximal inhibitory concentration (IC50) for each drug is indicated (mean± SD, n=2). D) Bar graphs showing growth rate of 22Rv1 cells treated with increasing doses of Indisulam (lef panel), Pladienolide B (middle panel) and THZ531 (right panel), evaluated as cell confluence ratio relative to time 0 (set to 1 and indicated by a red dashed line, mean± SD, n=3, two-way Anova). E) Representative images of the cytotoxicity assay performed using the IncuCyte® SX5 Live-Cell Analysis Imaging System on 22Rv1 cells treated with 3.3µM Ind, 10nM PladB and 200nM THZ531. Cells nuclei were labeled using the Nuclight Rapid NIR Dye (blue), dying cells were stained using the Incucyte® Cytotox Green Dye (green, indicated by the arrows in the images). F) Bar graph showing measures of the % of Cytotox Green Dye positive cells on the total number of cells using the Incucyte® Cell-by-Cell Analysis Software Module (mean ± SD, n=2, t-test, *=p <0.05, **p=<0.01). G,H) Western Blot (G) and densitometric analysis (H) of the expression levels of RBM39 in 22Rv1 cells treated or not (-) for indicated time with 3.3 µM indisulam. Actin was evaluated as loading control (mean ± SD, n=2, levels in DMSO treated cells were set to 1, *=p <0.05, ns= not significant).

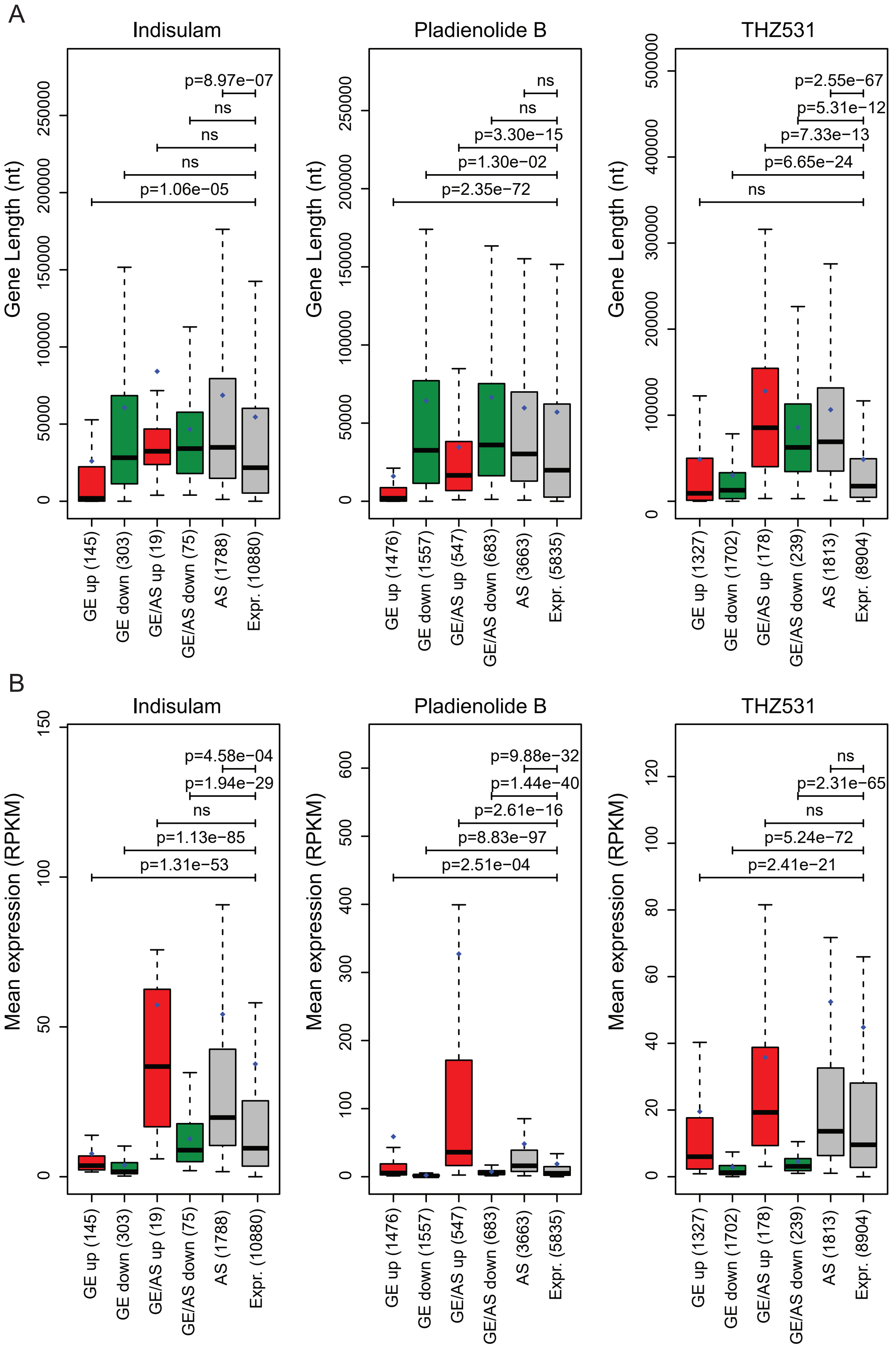

Supplementary Figure S2

Supplementary Figure S2. Specific structural characteristics and expression levels feature regulated genes in 22Rv1 cells line treated with Indisulam, Pladienolide B and THZ531. A, B) Box plots showing for each indicated treatment the gene length (A) and the RPKM values (B) of: genes up- (GE up) or down-regulated (GE down) exclusively at the expression level; genes regulated at splicing level and either up- (GE/AS up) or down-regulated (GE/AS down) at the expression level; genes regulated merely at the splicing level (AS) with comparison to other expressed and not regulated genes (Welch's t test).

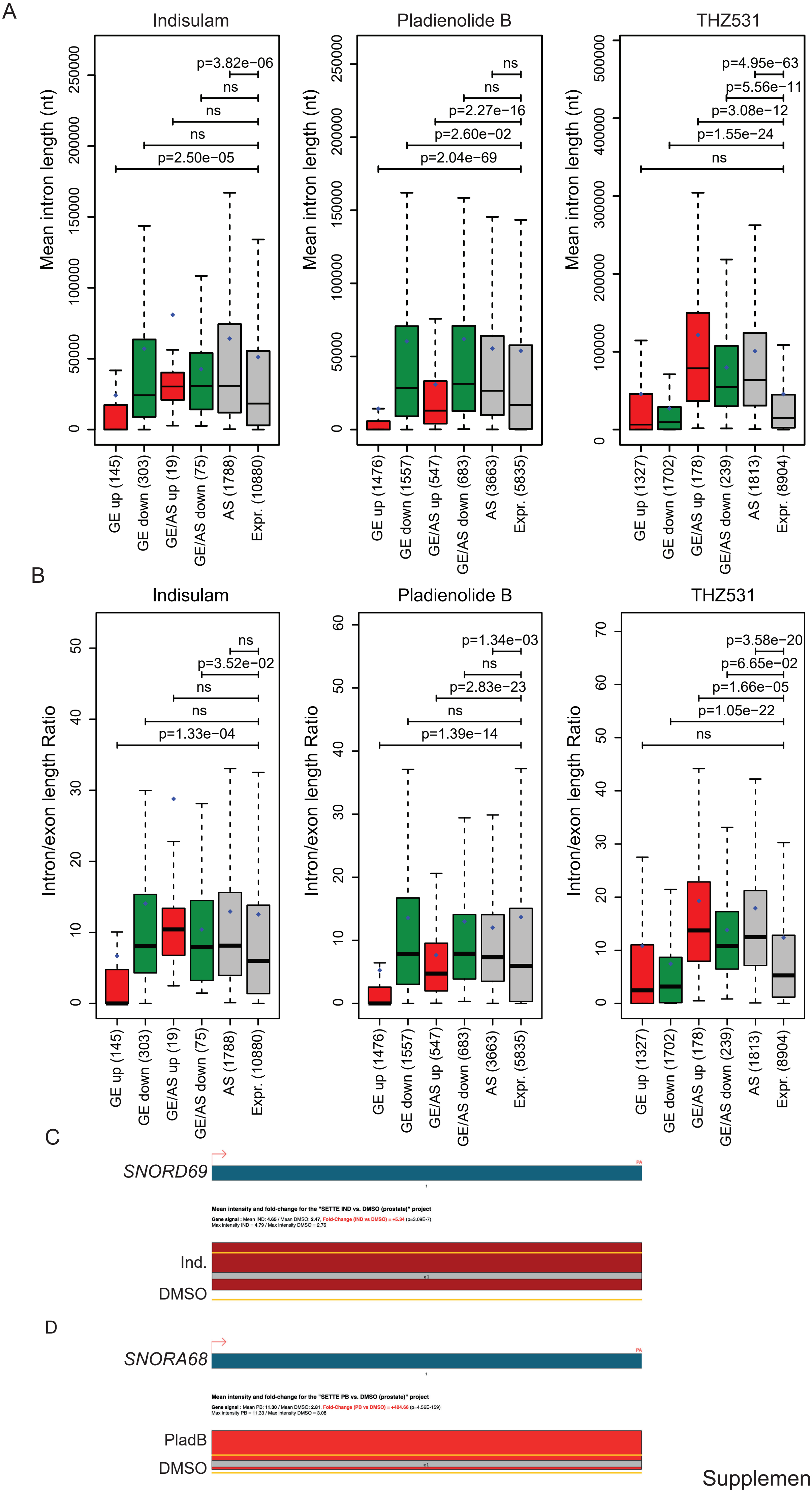

Supplementary Figure S3

Supplementary Figure S3. Shorter introns characterize up-regulated genes in PladB and Indisulam treated 22Rv1 cells. A, B) Box plots showing for each indicated treatment the mean intron length (A) and the intron/exon ratio (B) of: genes up- (GE up) or down-regulated (GE down) exclusively at the expression level; genes regulated at splicing level and either up- (GE/AS up) or down-regulated (GE/AS down) at the expression level; genes regulated merely at the splicing level (AS) with comparison to other expressed and not regulated genes (Welch's t test). C, D) Schematic visualization in the EASANA database of the RNA-seq reads for indicated intronless genes in 22Rv1 cells treated with either Indisulam (Ind, C) or Pladienolide B (Plad B, D) or the control vehicle (DMSO).

A

Category	Size
cassette for reference	924
constitutive exons for reference	3054
regulated cassette IND down	1538
regulated cassette IND up	130
regulated cassette PB down	1495
regulated cassette PB up	33
regulated cassette THZ down	260
regulated cassette THZ up	258

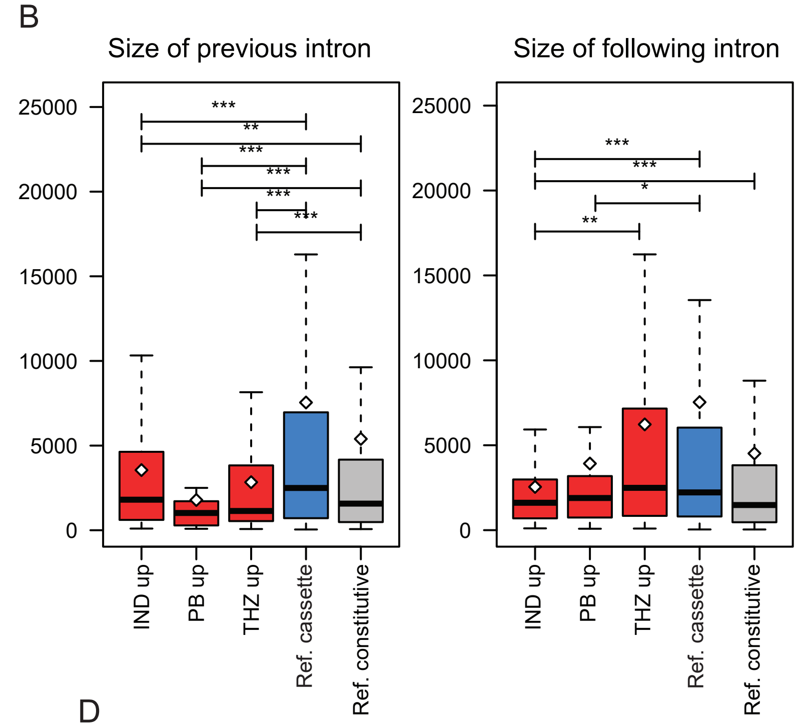

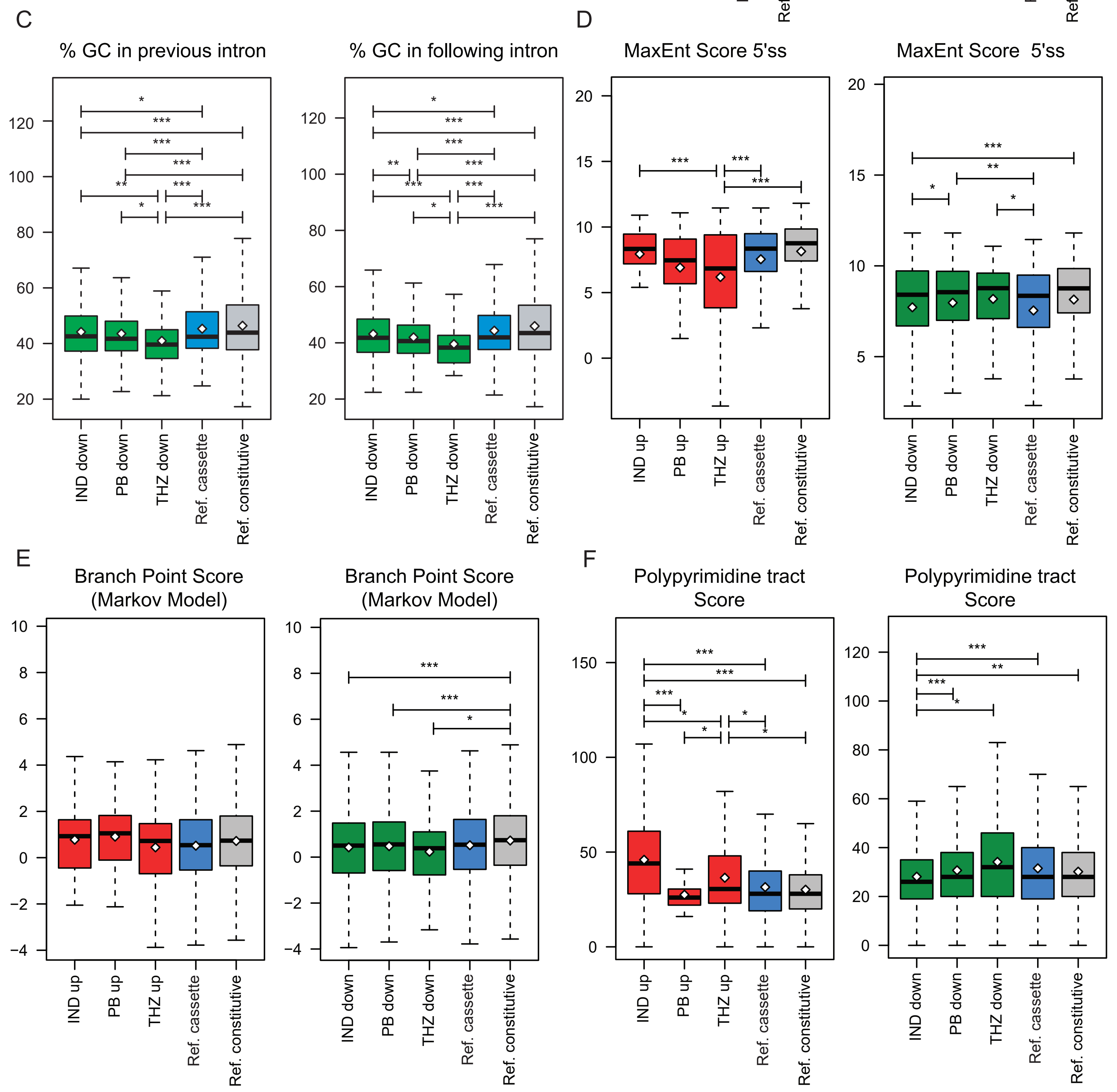

Supplementary Figure S4

Supplementary Figure S4. Shorter introns flank splicing-sensitive cassette exons to RNA splicing inhibitors in 22Rv1 cells. A) Table illustrating the number of exons within each group analysed. B-F) Boxplots showing comparison between up- (red boxes) or down-regulated exons (green boxes) by treatment with Indisulam (IND), Pladienolide B (PB) , THZ531 (THZ) and other not-regulated cassette exons (ref. cassette, blue box) and constitutive exons (ref. constitutive, grey box) for: the length of their flanking introns (B), % of GC content of their flanking introns (C), strength of their 5' splice-site (D), score of their branch-point (E) and score of their polypyrimidine tracks (F). Whiskers indicate 1.5 interquartile range and highlighted circles the mean values (*p ≤ 0.05, **p ≤ 0.01, ***p ≤ 0.001; ns = not significant, Welch's t test).

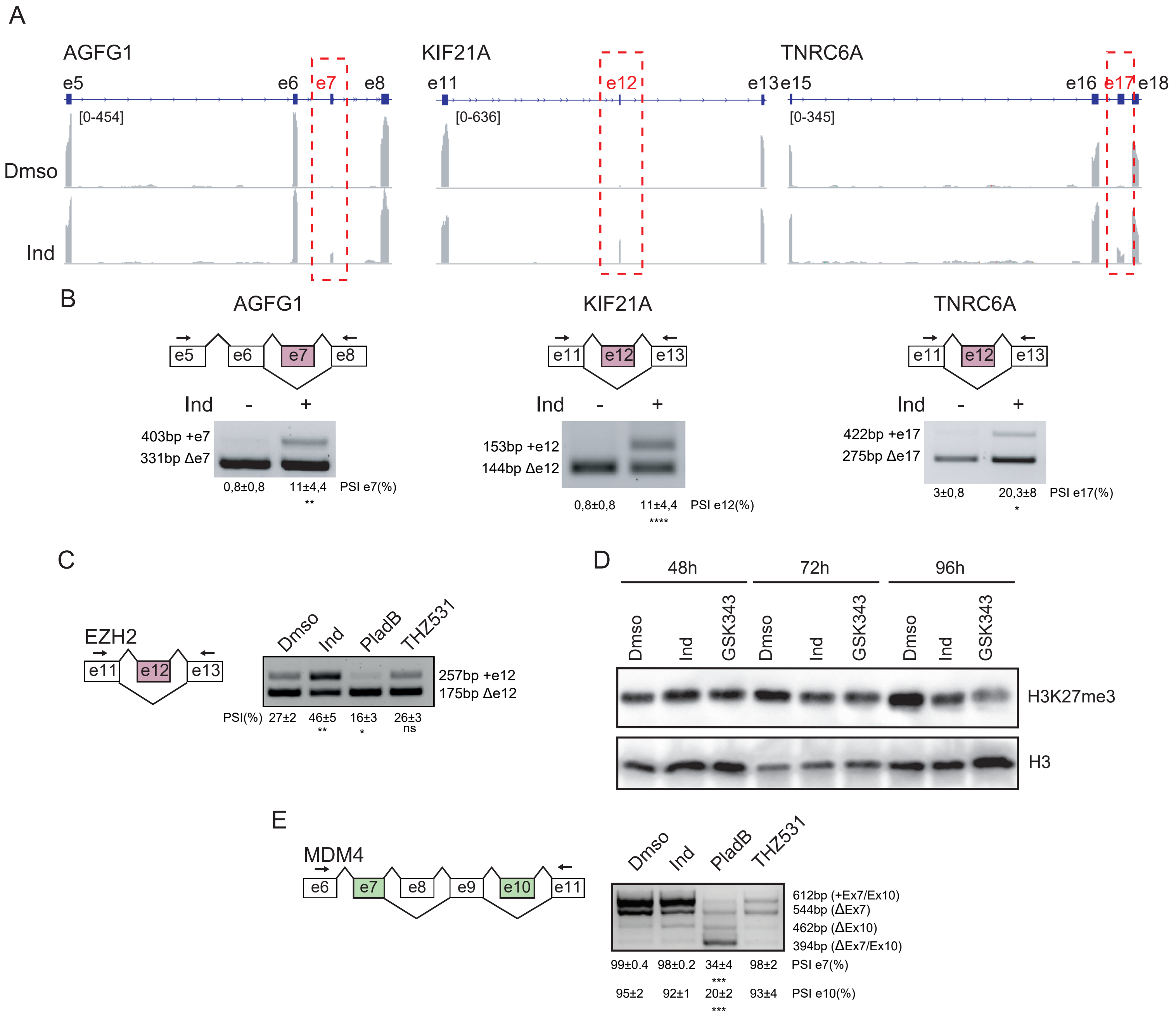

Supplementary Figure S5

Supplementary Figure S5. Inclusion of weak cryptic cassette exons is promoted by Indisulam treatment in 22Rv1. A) Profiles of the RNA-seq reads in 22Rv1 cells treated with Indisulam (Ind) or control vehicle (Dmso) of the genomic regions for the indicated genes encompassing the exon cassettes (dashed red boxes) sensitive to Ind and their flanking regions. B) Schematic representation of the indicated cassette exon splicing events and representative images of their RT-PCR analyses in 22Rv1 treated with Ind (3µM, 16hrs) or Dmso. Black arrows in the scheme indicate primers used for the PCR analysis. Results of the densitometric evaluation of the percentage of spicing inclusion (PSI) are shown below the agarose gel (mean ± SD, n = 3, p values were calculated according to one-way Anova. C) Schematic representation of the indicated cassette exon splicing events in the *EZH2* gene beside to the representative images of its RT-PCR analyses in 22Rv1 treated with Indisulam (Ind, 3µM, 16hrs), Pladienolide B (PladB, 10nM, 6hr), THZ531 (200nM, 6hr) or Dmso. Black arrows in the scheme indicate primers used for the PCR analysis. Results of the densitometric evaluation of the PSI are shown below the agarose gel (mean ± SD, n =2, one-way Anova). D) Representative Western Blot analysis of H3K27me3 levels in 22Rv1 cells treated for indicated time with vehicle (Dmso), Indisulam (Ind, 3µM) or GSK343 (10 µM). H3 was evaluated as loading control. E) Schematic representation of the indicated cassette exon splicing events in the *MDM4* gene beside to the representative images of its RT-PCR analyses in 22Rv1 treated with Indisulam (Ind, 3µM, 16hrs), Pladienolide B (PladB, 10nM, 6hr), THZ531 (200nM, 6hr) or Dmso. Black arrows in the scheme indicate primers used for the PCR analysis. Results of the densitometric evaluation of the PSI are shown below the agarose gel (mean ± SD, n =2, one-way Anova).

A

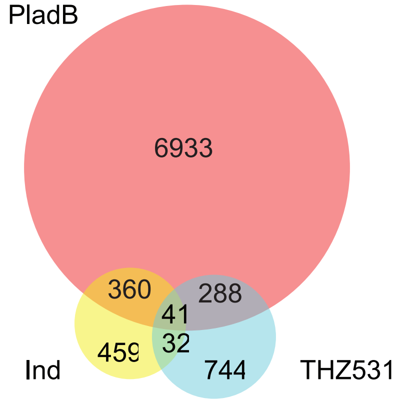

B

Category	Size
constitutive introns for reference	5982
intron retention for reference	232
regulated intron retention IND down	74
regulated intron retention IND up	844
regulated intron retention PB down	113
regulated intron retention PB up	7545
regulated intron retention THZ down	149
regulated intron retention THZ up	972

C

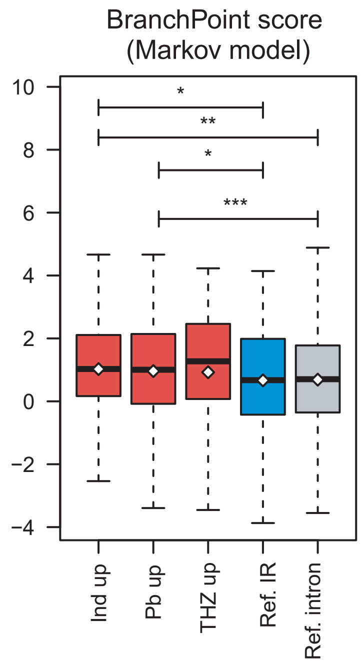

Supplementary Figure S6

Supplementary Figure S6. Pervasive intron retention is induced by splicing inhibitors in 22Rv1 cells. A) Overlap of regulated intronic events regulated by Indisulam (Ind), Pladienolide B (PladB) and THZ531 in 22Rv1 cells, according to RNA-seq data. B) Table illustrating the number of introns within each group analysed for cis-acting features shown in main Figure 5 and in C. C) Boxplot showing comparison between up-regulated introns (red boxes) by treatment with Indisulam (Ind), Pladienolide B (PB), THZ531 (THZ) and other not-regulated intron-retention events (ref. intron, blue boxes) and properly spliced-introns (ref. constitutive, grey box) for score of their branch-point (Whiskers indicate 1.5 interquartile range, Welch's t test).

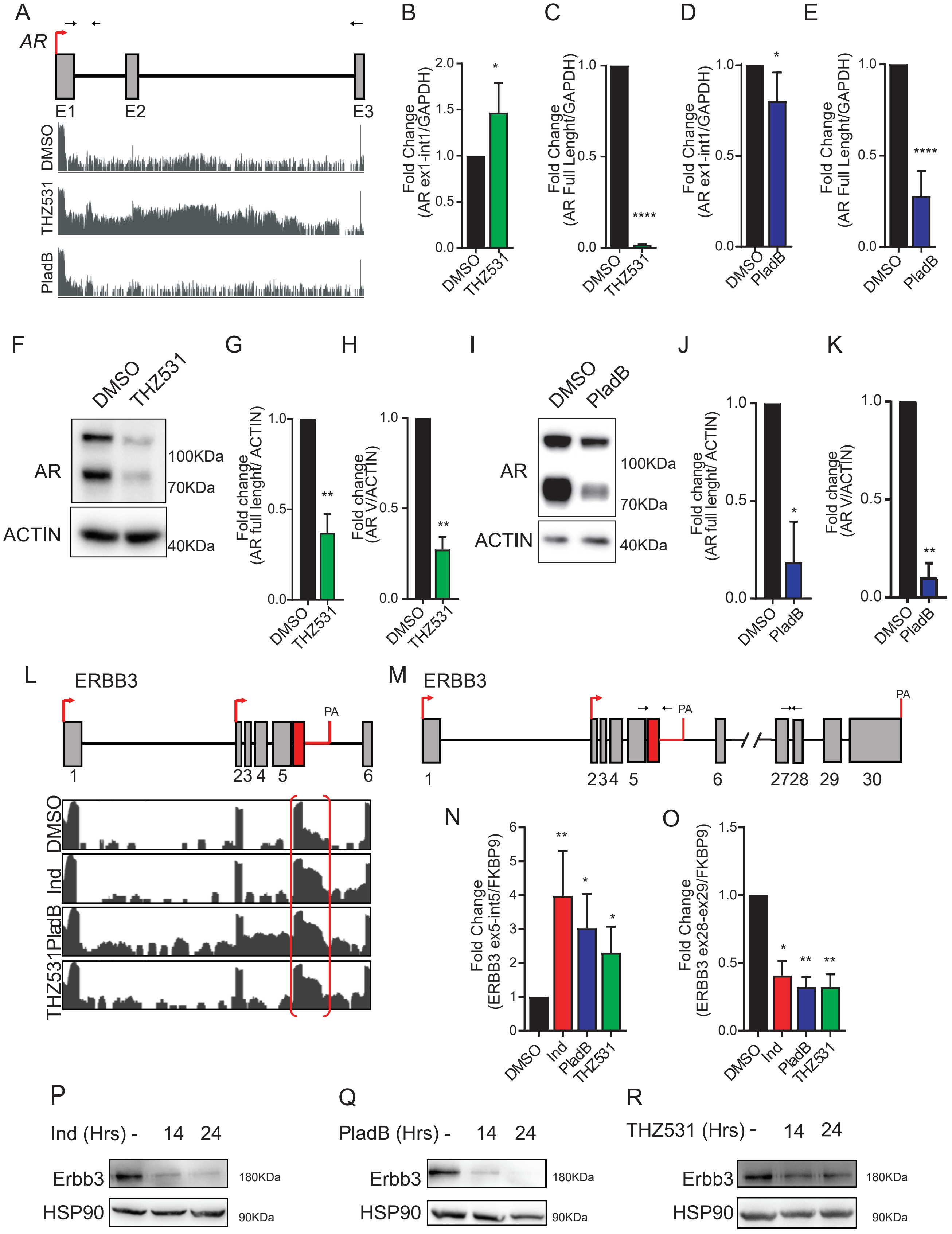

Supplementary Figure S7

Supplementary Figure S7. Splicing targeting drugs affect CRPC-relevant genes expression through intron retention and alternative last exon selection. A) Schematic representation and profile of RNA-seq reads of the genomic regions encompassing exon 1 to exon 3 of the *AR* gene in 22Rv1 cells treated with THZ531, PladB or control vehicle (DMSO). Black arrows indicate location of primers used for qPCR analysis in B-E). B-E) Bar graphs show the qPCR analysis for the relative ratio of AR ex1-int1/GAPDH (B,D) or AR Full length/GAPDH (C,E) in 22Rv1 cells treated with 100 μM THZ531 (B,C) or 10 nM PladB (D,E) with respect to those treated with DMSO (set to 1) (mean ± SD, n=3, t-test). F) Representative Western Blot images of AR protein expression in 22Rv1 cells treated for 24hrs with 100μM THZ531 or DMSO. ACTIN was evaluated as loading control. G,H) Bar graph showing the results of the densitometric analysis of the AR Full length (G) and AR-V (H) protein levels relative to ACTIN (represented as normalized ratio respect to DMSO treated cells, mean ± SD, n=3, t-test). I) Representative Western Blot images of AR protein expression in 22Rv1 cells treated for 24hrs with 10nM PladB or DMSO. ACTIN was evaluated as loading control. J,K) Bar graph showing the results of the densitometric analysis of the AR Full length (J) and AR-V (K) protein levels relative to ACTIN (represented as normalized ratio respect to DMSO treated cells, mean ± SD, n=3, t-test). L) Schematic representation and profile of RNA-seq reads of the genomic region encompassing exon 1 to exon 6 of *ERBB3* gene in 22Rv1 cells treated with Indisulam (Ind), Pladienolide B (PladB) THZ531 or control vehicle (Dmso). M) Schematic representation of ERBB3 transcript (from exon 1 to exon 6 and from exon 27 to exon 30) with black arrows indicating primers used for qPCR analysis in (N) and (O). N,O) Bar graphs showing the results of qPCR analysis of the relative ratio of ERBB3 ex5-int5/FKBP9 (N) or ex28-ex29/FKBP9 (O) in 22Rv1 cells treated with Indisulam, Pladienolide B and THZ531 with respect to those treated with DMSO (set to 1, mean ± SD, n=2, t-test). P-R) Representative Western Blot images of ERBB3 expression levels in 22Rv1 cells treated or not (-) with Ind (P), PladB (Q) or THZ531 (R) for indicated time. HSP90 was evaluated as loading control.

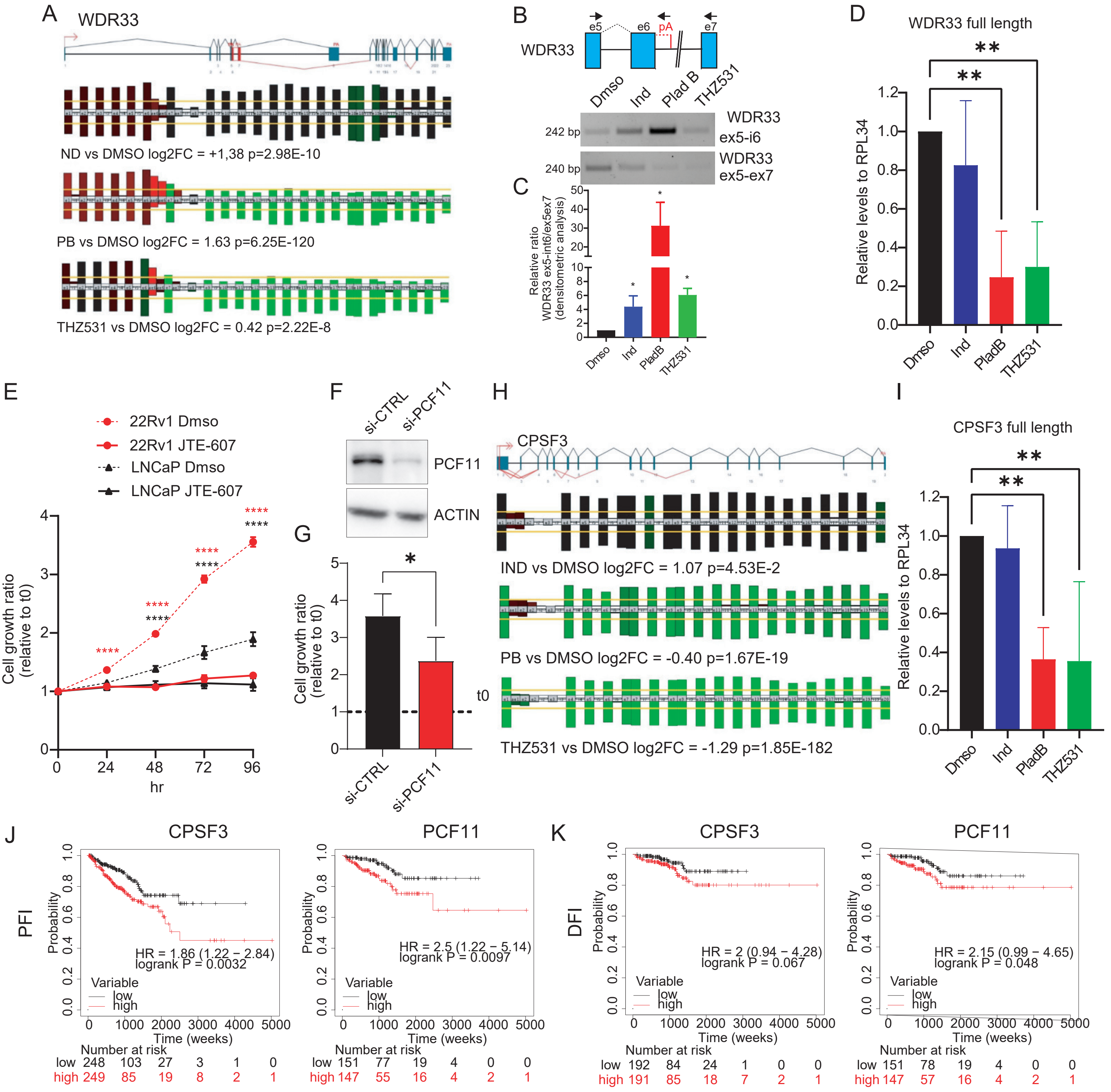

Supplementary Figure S8

Supplementary Figure S8. Splicing targeting drugs affect 3'-end mRNA processing related genes through intron retention and alternative last exon selection. A) Schematic visualization in the EASANA database of the RNA-seq reads for WDR33 gene in 22Rv1 cells treated with either Indisulam (Ind) or Pladienolide B (PB) or THZ531 with respect to the Dmso. Significance and log2 of the gene expression fold-change with respect to DMSO are indicated. B) RT-PCR analysis for WDR33 prematurely terminated or full-length transcripts. Exons, introns, polyadenylation (pA) signal and primers used in the PCR analysis are indicated in the scheme. C) Bar graph illustrating the results of the densitometric analysis of the ratio between WDR33 prematurely terminated and full-length transcript (mean ±SD n=3, t-test). D) qPCR analysis of WDR33 full length transcript relative levels to RPL34 in 22Rv1 cells treated with indicated drugs. Data are expressed as fold-change relative to Dmso treated cells (mean ± SD, n = 3, t-test). E) Line graph showing the growth rate of LNCaP and 22Rv1 cells treated with 25µM JTE-607or Dmso (mean ± SD, n = 3, two-way Anova). F) Western Blot analysis for PCF11 protein levels in 22Rv1 after 48h of transfection with 40nM of indicated siRNAs. ACTIN was evaluated as loading control. G) Bar graph showing the growth rate of 22Rv1 cells at 96h after transfection with indicated siRNAs (mean ± SD, n = 3, t-test). H) Schematic visualization in the EASANA database of the RNA-seq reads for CPSF3 gene in 22Rv1 cells treated with either Indisulam (Ind) or Pladienolide B (PB) or THZ531 with respect to the DMSO. Significance and log2 of the gene expression fold-change with respect to DMSO are indicated. I) qPCR analysis of CPSF3 full length transcript relative levels to RPL34 in 22Rv1 cells treated with indicated drugs. Data are expressed as fold-change relative to Dmso treated cells (mean ± SD, n = 3, t-test). J,K) Kaplan Meier graphs of the progression free interval (J) and disease free interval (K) survival probability of prostate carcinoma patients in the TCGA database classified according to the median levels of CPSF3 or PCF11 genes.
